# Distinct adhesion-independent functions of β-catenin control stage-specific sensory neurogenesis and proliferation

**DOI:** 10.1186/s12915-015-0134-4

**Published:** 2015-04-11

**Authors:** Max Hans-Peter Gay, Tomas Valenta, Patrick Herr, Lisette Paratore-Hari, Konrad Basler, Lukas Sommer

**Affiliations:** Cell and Developmental Biology Division, Institute of Anatomy, University of Zurich, Winterthurerstrasse 190, 8057 Zurich, Switzerland; Institute of Molecular Life Sciences, University of Zurich, Winterthurerstrasse 190, 8057 Zurich, Switzerland; Present address: SciLifeLab, Stockholm, Sweden; Present address: University Hospital Zurich, Clinical Trials Center, Zurich, Switzerland

**Keywords:** Sensory neurogenesis, Cell fate decisions, Cellular proliferation, Canonical Wnt signaling, Signaling versus adhesion function of β-catenin

## Abstract

**Background:**

β-catenin plays a central role in multiple developmental processes. However, it has been difficult to study its pleiotropic effects, because of the dual capacity of β-catenin to coordinate cadherin-dependent cell adhesion and to act as a component of Wnt signal transduction. To distinguish between the divergent functions of β-catenin during peripheral nervous system development, we made use of a mutant allele of β-catenin that can mediate adhesion but not Wnt-induced TCF transcriptional activation. This allele was combined with various conditional inactivation approaches.

**Results:**

We show that of all peripheral nervous system structures, only sensory dorsal root ganglia require β-catenin for proper formation and growth. Surprisingly, however, dorsal root ganglia development is independent of cadherin-mediated cell adhesion. Rather, both progenitor cell proliferation and fate specification are controlled by β-catenin signaling. These can be divided into temporally sequential processes, each of which depends on a different function of β-catenin.

**Conclusions:**

While early stage proliferation and specific Neurog2- and Krox20-dependent waves of neuronal subtype specification involve activation of TCF transcription, late stage progenitor proliferation and Neurog1-marked sensory neurogenesis are regulated by a function of β-catenin independent of TCF activation and adhesion. Thus, switching modes of β-catenin function are associated with consecutive cell fate specification and stage-specific progenitor proliferation.

**Electronic supplementary material:**

The online version of this article (doi:10.1186/s12915-015-0134-4) contains supplementary material, which is available to authorized users.

## Background

Canonical Wnt signaling is one of the most important evolutionarily conserved signaling pathways in embryonic development [1-3]. There are 19 Wnt genes found in mammals and a multitude of receptors to which they bind. In the canonical Wnt signaling pathway the interaction between Wnt and the transmembrane receptor proteins Frizzled and LRP leads to recruitment of Axin and GSK-3 to the membrane, thus preventing degradation of β-catenin. This in turn results in the accumulation of β-catenin in the cytoplasm and its translocation into the nucleus, where it can interact with one of the members of the TCF/Lef family. In the absence of β-catenin, TCF/Lef transcription proteins act as transcriptional repressors by binding to Groucho/TLE co-repressors. Nuclear β-catenin physically displaces Groucho co-repressors and converts TCF/Lef into transcriptional activators [4]. In recent years, evidence has revealed that the purpose of some TCFs is not only their function to serve as trans-activators, but also their ability to act as repressors when bound by Groucho/TLE co-repressors. In this case, TCF targets become de-repressed upon removal of Groucho/TLE co-repressors due to the binding of nuclear β-catenin. The main TCF prone to act as a repressor is TCF3, whereas TCF4 and TCF1 have the capacity to fulfill both activator and repressor functions depending on the spatial and temporal context [5-12].

**Figure 1:**
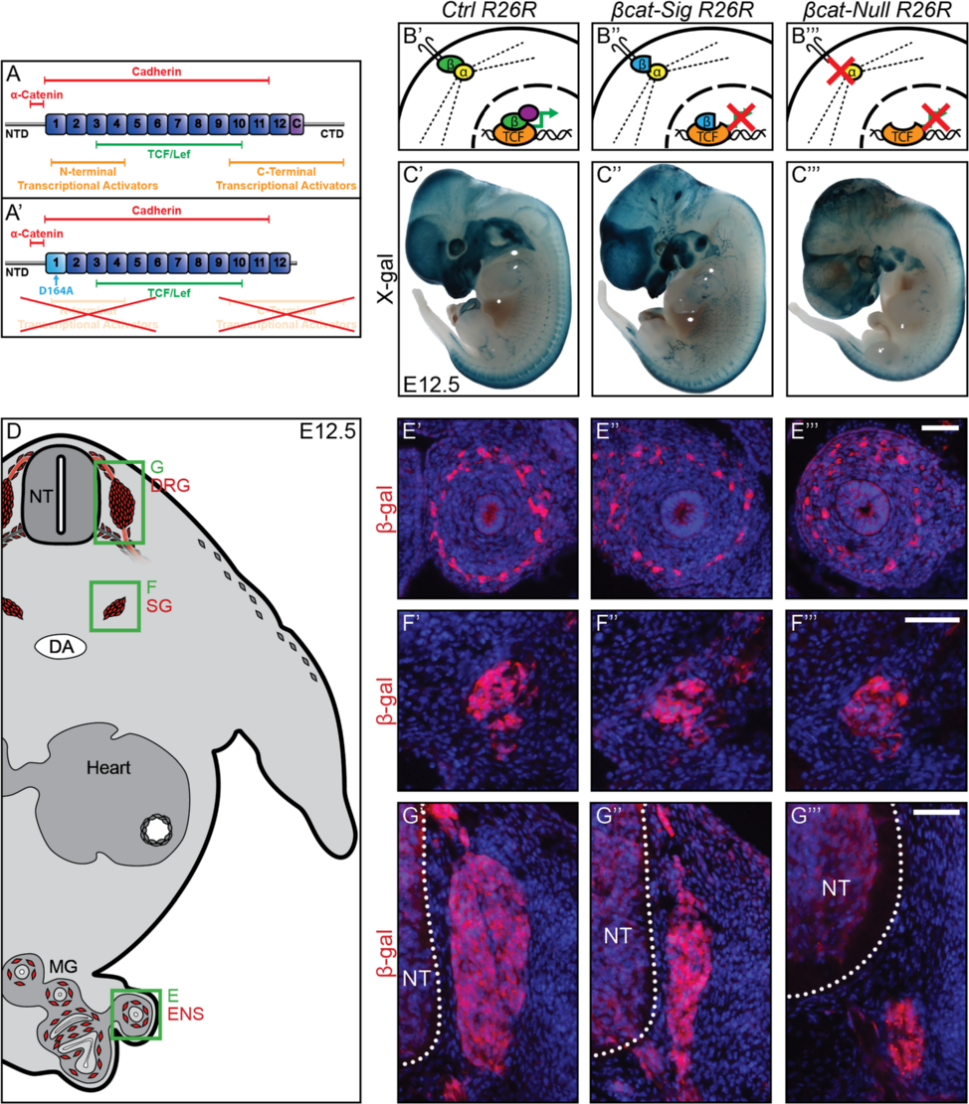
Total loss of β-catenin leads to a more severe phenotype in the dorsal root ganglia than inhibition of the TCF/Lef transcriptional output of β-catenin. **(A)** The β-catenin protein consists of 12 Armadillo repeats (numbered boxes), a conserved helix-C (C), an amino-terminal domain (NTD), and a carboxy-terminal domain (CTD) [15]. Colored bars show binding sites for β-catenin interaction partners: red, components of adherens junctions; green, TCF/Lef transcription factors providing DNA binding; orange, transcriptional co-activators. **(A’)** Diagram presenting β-catenin-dm. D164A and truncation of CTD inhibits association with multiple players of the transcription machinery. However, binding sites for TCF/Lef and components of adherens junctions are preserved. **(B’-B”’)** Schematic of the functional properties provided by the distinct β-catenin alleles. **(B’)** β-catenin (green) of control animal induces transcription (green arrow) by binding with TCF/Lef (orange) in the nucleus and recruiting co-transcription factors (purple). Furthermore, it links transmembrane cadherins via α-catenin (yellow) to the actin cytoskeleton (dotted line). **(B”)** β-catenin-dm (blue) inhibits TCF/Lef-mediated transcription, but preserves cadherin-mediated adhesion. **(B”’)** Cells of *βcat-Null* animals lose both TCF/Lef-mediated transcription and cadherin-mediated adhesion. **(C’-C”’)** *In vivo* fate mapping of *Wnt1-Cre* embryos carrying the *R26R* reporter allele at E12.5. **(D)** Illustration of a transverse section of an E12.5 control animal displaying in red the neural derivatives of neural crest cells: dorsal root ganglia (DRG); sympathetic ganglia (SG); enteric nervous system (ENS). Green boxes display caption area for subfigures E-G. **(E’-G”’)** Immunohistochemistry for β-gal on transverse sections. Normal development of the ENS **(E’-E”’)** and the SG **(F’-F”’)** can be witnessed in both mutants at E12.5. **(G’-G”’)**. The size of the DRG of *βcat-Sig R26R* embryos is strongly reduced **(G”)**, whereas DRG of *βcat-Null R26R* animals are virtually non-existent (**G”’)**. NT, neural tube; DA, dorsal aorta; MG, mid gut. Scale bars: 50 μm. E, embryonic day.

β-catenin is the bottleneck of canonical Wnt signaling, as it is the central, non-redundant component of the pathway. This trait has made β-catenin especially attractive as a target for genetic manipulation to elucidate the impact of Wnt signaling *in vivo*. The β-catenin protein is composed of 12 Armadillo repeats and is flanked by an amino-terminal domain and a conserved helix-C next to a carboxy-terminal domain. Nuclear β-catenin interacting with TCF/Lef recruits transcriptional co-activators to the transcription complex by means of its N- and C-terminal, initiating TCF/Lef-mediated transcription [13] (Figure 1A). However, apart from its role as a transcriptional co-activator, β-catenin also serves as a linking protein in cadherin-mediated adherens junction. In adherens junctions, transmembrane cadherins bind to the first eleven Armadillo repeats of β-catenin, while actin-bound α-catenin binds to the amino-terminal domain. This dual role of β-catenin in mediating Wnt signaling and cell-cell adhesion made it difficult to collate a specific phenotype obtained upon β-catenin gene (*Ctnnb1*) manipulation to either or both functions of β-catenin. To distinguish between signaling and adhesion functions of β-catenin in the skin, a transgene has previously been used [14], which led to expression of a mutated form of β-catenin harboring a C-terminal truncation and lacking the first 87 amino acids. However, this version of β-catenin was non-degradable, unlike its physiological counterpart, and did not block interaction with N-terminal co-activators. We recently generated another allele of β-catenin (*Ctnnb1*^*dm*^) that results in expression of a mutated form of the protein from its endogenous locus. The protein expressed from this allele (β-catenin-dm) is degradable in the absence of Wnt, is fully functional in adherens junctions, and lacks known signaling properties as a transducer of Wnt-dependent transcriptional activation [15,16]. Moreover, a dominant negative effect of the mutated protein can be excluded as heterozygous animals carrying *Ctnnb1*^*dm*^ display no phenotype [15]. This form of β-catenin has a single amino acid change in the first Armadillo repeat of β-catenin (D164A), which prevents the binding of the N-terminal transcriptional co-activators BCL9/BCL9L. These regulators are important independently of C-terminal co-activators. Indeed, homozygosity of the D164A mutation leads to lethality in mouse embryos at embryonic day (E10.5) [15]. Additionally, a truncation of its C-terminus blocks the association of β-catenin-dm with a multitude of co-activators acting as chromatin modifiers (CBP/p300, Brg1) or connecting β-catenin to RNApolII machinery (Paf1 complex, MEDIATOR complex). Importantly, β-catenin-dm is still able to bind to cadherins, α-catenin, and TCF/Lef (Figure 1A’). Therefore, β-catenin-dm maintains the ability to mediate cellular adhesion and, likely, to de-repress TCF targets, allowing the identification of effects of β-catenin that are TCF-transactivation independent.

Neural crest cells (NCCs) are a population of multipotent cells that delaminate from the dorsal part of the neural tube during neurulation of vertebrate embryos [17]. Upon delamination, NCCs migrate along specific routes throughout the embryo to give rise to a broad variety of derivatives, such as the neuronal and glial cells of the peripheral and enteric nervous system as well as craniofacial bone, cartilage, smooth muscle, and melanocytes. Wnt signaling has been implicated at multiple developmental stages of the neural crest [18-24]. In particular, we have previously demonstrated the consequences of conditional ablation of β-catenin in the premigratory NCCs using *Cre* recombinase driven by the Wnt1 promotor (*Wnt1-Cre*) [18,25]. Inactivation of β-catenin in *Wnt1-Cre Ctnnb1*^*flox/flox*^ embryos resulted in a drastic reduction of sensory neuronal, and complete absence of glial, lineages in the dorsal root ganglia (DRG), whereas other neural derivatives, such as sympathetic ganglia and the enteric nervous system, appeared to develop normally [18]. Sensory neurogenesis involves the generation of multiple neuronal subtypes in three temporal waves and is tightly associated with the aggregation of NCCs in DRG [26]. However, it remains to be determined to what extent β-catenin transactivation signaling as opposed to other functions, such as β-catenin-mediated adhesion and TCF/β-catenin-mediated de-repression, controls sensory neuronal subtype specification and DRG formation. Here, we exploited our novel β-catenin signaling mutant allele, *Ctnnb1*^*dm*^, together with β-catenin null and other conditional knockout alleles, and reveal distinct requirements of β-catenin functions for sensory neurogenesis. Surprisingly, while β-catenin/α-catenin-mediated cellular adhesion plays a negligible role in sensory neuron formation, several steps in sensory neurogenesis require β-catenin, but only some of these are dependent on β-catenin-mediated transcriptional activation.

## Results

### Replacement of β-catenin with its signaling-defective variant in neural crest stem cells exhibits milder phenotypes in comparison to complete ablation of β-catenin

To investigate the role of β-catenin signaling during neural crest development independently of β-catenin-mediated cell adhesion, we generated *Wnt1-Cre Ctnnb1*^*flox/dm*^ (*βcat-Sig*) animals. In these mice, Cre-mediated conditional deletion of β-catenin normally expressed from the *Ctnnb1*^*flox*^ allele leads to complete replacement of the endogenous β-catenin protein by the signaling mutant form derived from the *Ctnnb1*^*dm*^ allele (Figure 1A’; [15]). As a result, *Wnt1-Cre*-positive premigratory NCCs and their derivatives express mutant β-catenin that retains the cell adhesion function, but fails to activate TCF/Lef-dependent transcription (Figure 1B”). For comparison, we also produced *Wnt1-Cre Ctnnb1*^*flox/flox*^ (*βcat-Null*) embryos (Figure 1B”’). Furthermore, to perform *in vivo* cell lineage tracing in both mouse models, we made use of the ROSA26 Cre reporter line (*R26R*) [27], which expresses β-galactosidase (*β-gal*) upon Cre-mediated recombination.

X-gal staining at E 12.5 on whole-mount embryos carrying *R26R* revealed similar, but less severe, phenotypes in *βcat-Sig R26R* embryos when compared to *βcat-Null R26R* embryos (Figure 1C’-C”’). Craniofacial and mesencephalic defects were the most obvious macroscopic phenotype in both mutant embryos when compared to control embryos, as previously described [15]. Immunohistochemistry for *Cre*-dependent β-gal expression on transverse sections of E12.5 embryos revealed unaffected development of the enteric nervous system and the sympathetic ganglia in either mutant embryo (Figure 1E’-E”’, F’-F”’), in agreement with [18]. In contrast, proper formation of the DRG was inhibited in E12.5 *βcat-Null R26R* embryos as evident by a reduction of DRG-populating cells by 91.7 ± 0.6% (Figure 1G”’), while the cell number in *βcat-Sig R26R* DRG was reduced by 74.9 ± 3.8% (Figure 1G”).

The lineage-specific phenotypes observed in *βcat-Sig R26R* and *βcat-Null R26R* embryos might reflect distinct canonical Wnt signaling activity in derivatives of NCCs. Functional output of β-catenin was assessed using the *LacZ* expressing Wnt-reporter line *BAT-gal* [28]. In these mice, *Cre*-induced enhanced green fluorescent protein (EGFP) expression was used to be able to trace recombined *BAT-gal* positive cells [29]. Double immunofluorescent staining for β-gal and EGFP on transverse sections of E10.5 embryos revealed that NCCs giving rise to progenitors of the cardiac outflow tract, the enteric nervous system, and the sympathetic ganglia, were not subjected to canonical Wnt signaling post migration (Additional file 1: Figure S1A-C). However, EGFP-positive cells populating the pharyngeal arches (Additional file 1: Figure S1D) and DRG (Additional file 1: Figure S2B) strongly expressed the output of the *BAT-gal* reporter.

To verify the loss of wild type β-catenin in our mutant mouse models, antibodies specific for either the N- or C-terminus of β-catenin were used for immunohistochemistry. Triple immunofluorescent staining for EGFP and the N- and C-terminus of β-catenin on transverse sections of E10.5 embryos confirmed the loss of β-catenin in recombined cells of *βcat-Null* animals and substitution of wild type β-catenin by the double mutated form with a truncated C-terminus present in recombined cells of *βcat-Sig* mutants (Additional file 1: Figure S2E’-G””). Moreover, on adjacent sections, double immunofluorescent staining for EGFP and β-gal of the *BAT-gal* reporter demonstrated the loss of Wnt/β-catenin-induced TCF/Lef-mediated transcription (Additional file 1: Figure S2B-D).

### Postmigratory proliferation of dorsal root ganglia precursors is temporally regulated by different functions of β-catenin

**Figure 2:**
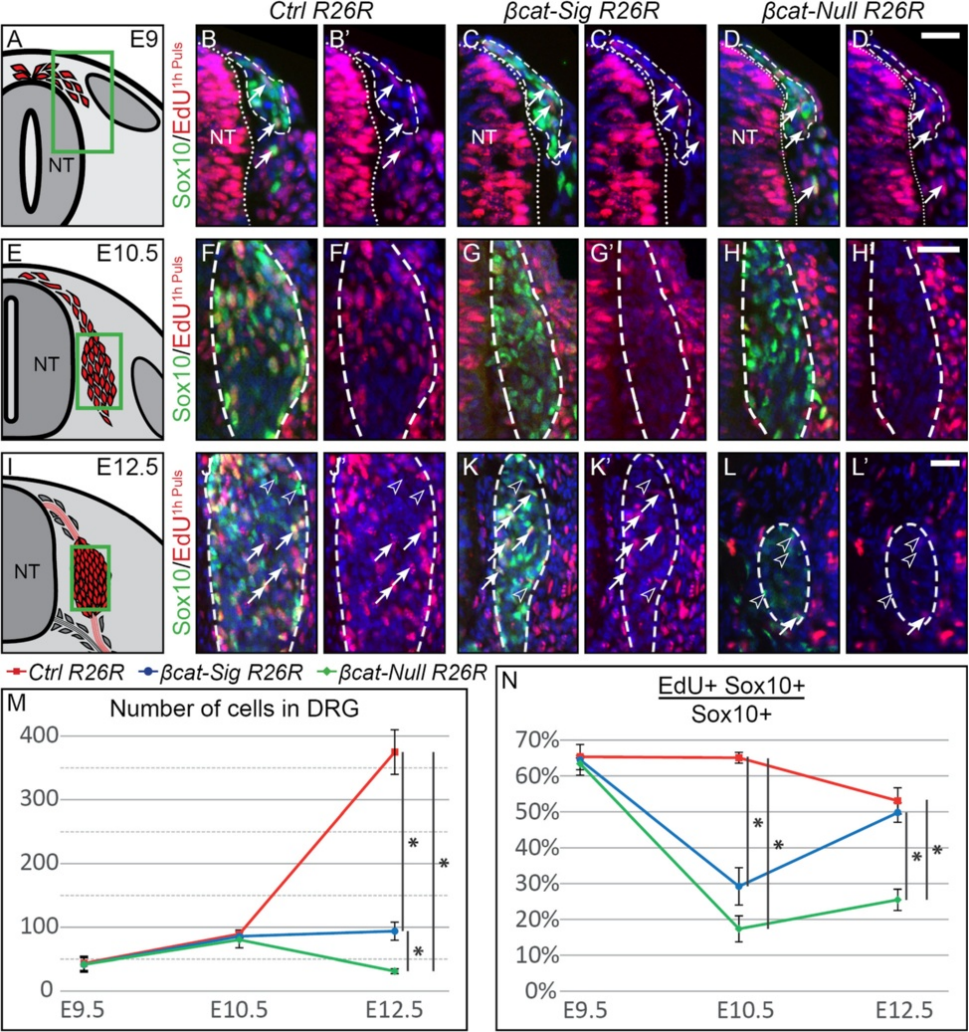
Rescue of proliferation defects in dorsal root ganglia precursors of βcat-Sig embryos. **(A, E, I)** Illustration of a transverse section at E9, E10.5, and E12.5, respectively, displaying cells contributing to the DRG in red. Green box represents caption area for subfigures B-D, F-H and J-L, respectively. **(B-D, F-H, J-L)** Immunofluorescent staining for Sox10 and detection of EdU from a one-hour pulse. Dashed lines frame cells of the *R26R* reporter fate mapped *in vivo*, which were detected by an immunofluorescent co-staining for β-gal on the corresponding sections. **(B-D, B’-D’)** No difference in the amount of Sox10- and EdU- double positive cells is apparent between the three types of embryos at E9. Arrows: Sox10 and EdU co-expressing cells. **(F-H, F’-H’)** At E10.5, both mutant animals display reduced incorporation of EdU in Sox10-positive DRG progenitors. **(J-L, J’-L’)** However, Sox10-positive cells of *βcat-Sig R26R* embryos recover the ability to incorporate EdU at E12.5, while Sox10-positive cells of *βcat-Null R26R* embryos of the same age do not. Arrows: Sox10 and EdU co-expressing cells, Arrowheads: Sox10-positive EdU-negative cells. **(M)** Quantification of the average numbers of *R26R*-positive cells contributing to the DRG at E9, E10.5 and E12.5 reveals no significant differences between mutant and control animals at E9 and E10.5. However, at E12.5 there is a statistically significant decrease of cells populating the DRG in both mutant animals. The decrease is significantly stronger in *βcat-Null R26R* embryos than in *βcat-Sig R26R* embryos. **(N)** Percentages of EdU-positive cells in the Sox10-positive population display a significant reduction of EdU incorporation in *βcat-Null R26R* animals at E10.5 and E12.5. Whereas *βcat-Sig R26R* animals exhibit similar statistically significant reduction as *βcat-Null R26R* animals at E10.5, they recover the ability to incorporate EdU by E12.5. NT, neural tube; * indicates *P* <0.05; Scale bars: 25 μm. DRG, dorsal root ganglia; E, embryonic day; EdU, 5-ethynyl-2′-deoxyuridine.

To determine the cause for the reduction of the sensory lineage in *βcat-Sig* and *βcat-Null* embryos, respectively, the presence of undifferentiated DRG progenitors during migration at E9.5 and post migration at E10.5 and E12.5 was analyzed by immunohistochemistry for the transcription factor Sox10 expressed by the multipotent NCCs [30] and β-gal expressed from the *R26R* lineage tracer (Figure 2B-D, F-H, J-L). In comparison to control embryos, the number of Sox10-positive progenitors was retained in both mutants in migratory NCCs at E9.5 as well as in forming DRG at E10.5 (Figure 2B-D, F-H). The reduction of the DRG size in both mutant embryos relative to control DRG was first observed after E10.5. Interestingly, from E10.5 to E12.5 the DRG of the *βcat-Sig R26R* embryos maintained a relatively constant size, whereas the size of the DRG of the *β-cat-Null R26R* mutants began to decrease (Figure 2M).

It is well known that sensory neurons undergo apoptosis as of E11 [11]. However, a normal distribution of apoptotic cells was observed in mutant DRG and their progenitors from E9 to E12.5, as revealed by staining for cleaved caspase 3 (data not shown). The presence of DRG progenitors and the exclusion of irregular apoptosis suggested a proliferation defect as cause for the reduced DRG size in both mutants. Unexpectedly, the expression of KI67, a marker for all phases of the cell cycle, was unchanged in both mutants at any time point from E9 to E12.5 (data not shown). Wnt signaling can coordinate cell cycle progression by regulating modulators of cell cycle checkpoints [31]. To investigate proper cell cycle progression, we labeled DRG progenitors entering or progressing through S-phase with a one hour EdU pulse. In migratory NCCs at E9, the ratio of EdU/Sox10-double positive cells per all Sox10-positive cells was similar in all three genotypes (Figure 2B’-D’, N). However, at E10.5 postmigratory Sox10 and EdU co-expressing DRG progenitors were significantly reduced in both mutant embryos (Figure 2F’-H’, N). Interestingly, in *βcat-Sig R26R* embryos, the percentage of EdU-positive cells in the Sox10-positive population started to recover after E10.5 and was rescued at E12.5 (Figure 2J’-L’, N). Taken together, we observed in both mutants a decrease in DRG progenitors correctly progressing through the cell-cycle at E10.5 This phenotype was maintained in *βcat-Null* embryos at later stages, whereas in *βcat-Sig* embryos mitotically active DRG progenitors slowly regained proper cell cycle progression over time and recuperated at E12.5. The loss of proper cell cycle progression in *βcat-Null* animals is probably the reason for the loss of DRGs in these animals at later developmental stages [18]. In contrast, DRGs were detectable in *βcat-Sig* embryos at E16.5, although they were smaller than those of control animals (Additional file 1: Figure S3). Presumably, this phenotype was due to a reduction of the DRG progenitor pool at earlier stages, even though cell cycle progression was rescued in the *βcat-Sig* animals from E12.5 onwards. This suggests that during early stages of DRG development normal proliferation of DRG progenitors depends on β-catenin as a co-transcription factor of TCF/Lef-mediated transcription. As time progresses, regulators of correct cell cycle progression apparently start to depend on an alternative function of β-catenin independent of mediating TCF/Lef transcription.

### Diverse functions of β-catenin are required for subtype specification of the three waves of sensory neurogenesis

In peripheral sensory lineages, neuronal subtype specification occurs in sequential waves of neurogenesis driven by proneural transcription factors [26]. The basic helix-loop-helix (bHLH) transcription factor neurogenin 2 (Neurog2) initiates a first wave of neurogenesis and is expressed in an early population of migratory NCCs. It is continuously expressed throughout their migration until they reach the forming DRG, after which it is largely down regulated [32,33]. Migratory cells expressing Neurog2 at high levels give rise to tyrosine receptor kinase (Trk) B- and TrkC-positive neurons [32]. A later wave of neurogenesis is formed by Sox10-positive DRG progenitors, of which a subgroup will express the bHLH transcription factor neurogenin 1 (Neurog1) post migration within the coalesced DRG. This Neurog1-mediated wave mainly gives rise to neurons expressing TrkA [32], but can partially contribute to the TrkB and TrkC population. Furthermore, both waves of DRG progenitors generate glia. The third and final wave of neurogenesis produces the boundary cap cells (BCCs). BCCs give rise to peripheral glia, which appear in small clusters at the surface of the spinal cord, at prospective motor exit points and dorsal root entry zones. A fraction of cells surrounding the dorsal root entry zone migrate into the formed DRG and generate a small population of TrkA-positive neurons [34].

**Figure 3:**
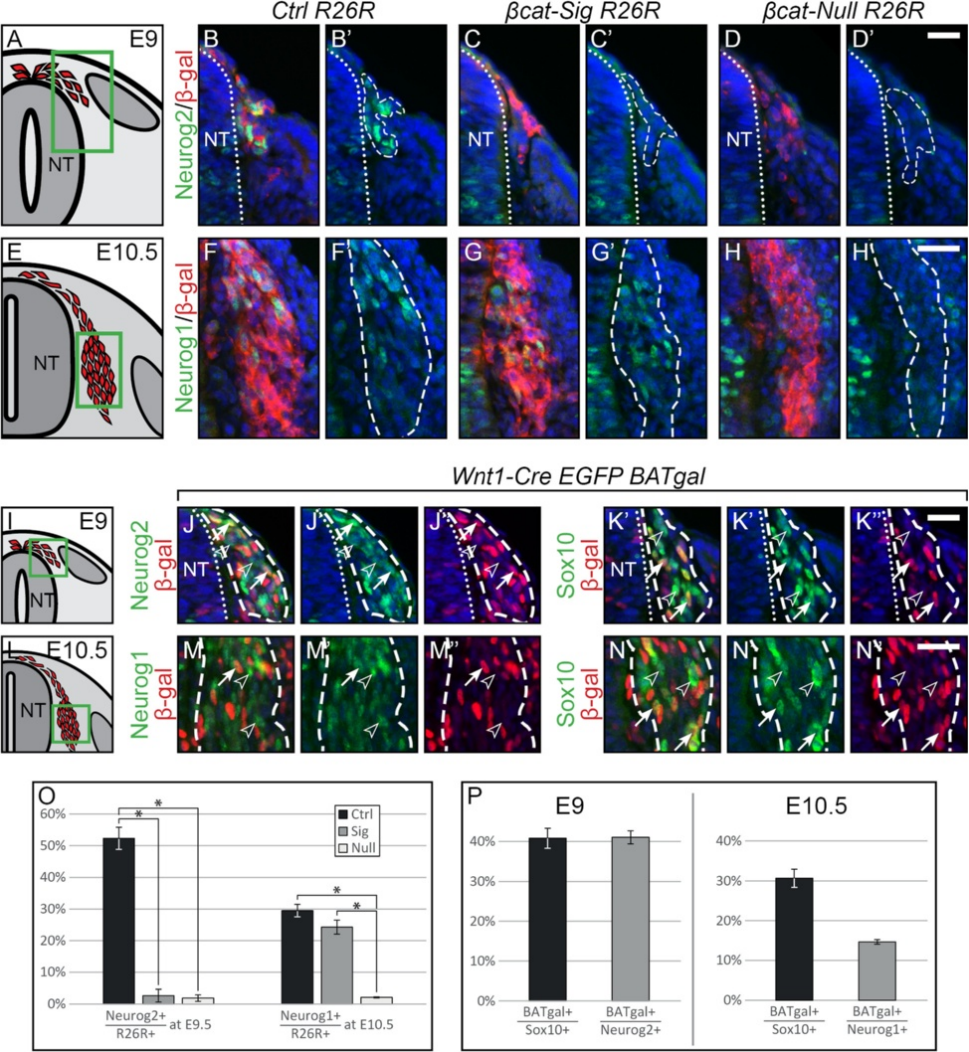
The first two waves of sensory neurogenesis depend on β-catenin. **(A, E, I, L)** Illustration of a transverse section at E9 and E10.5, respectively, displaying cells, which contribute to the DRG in red. Green box represents caption area for subfigures B-D, F-H, J-K and M-N, respectively. **(B-D, G-H)** Double immunohistochemistry for β-gal expressed by the *R26R* reporter and the sensory neurogenesis transcription factors Neuog2 at E9 or Neurog1 at E10.5, respectively. **(B’-D’)** Migrating DRG progenitors do not express Neurog2 in either of the two mutant types. **(F’-H’)** Whereas *βcat-Null R26R* animals lose expression of Neurog1 in DRG progenitors, progenitors of *βcat-Sig R26R* animals maintain expression of Neurog1. **(J-K, M-N)** Double immunohistochemistry for β-gal expressed by the *BAT-gal* Wnt-reporter and the sensory neurogenesis transcription factors Neurog2 at E9 or Neurog1 at E10.5 as well as Sox10 at both stages, respectively. **(J’-J”, K’-K”, M’-M”, N’-N”)** corresponding single fluorescence channels of the sections shown in J, K, M and N, respectively. Arrows indicate double positive cells, arrowheads indicate BAT-gal-negative cells within the stained subpopulations. **(O)** Quantification of Neurogenin-positive cells per *R26R*-positive cells contributing to the DRG at E9 and E10.5 show an almost complete loss of Neurog2 in both mutants at E9 and a significant reduction of Neurog1 in the *βcat-Null R26R* mutant at E10.5. **(P)** Percentage of *BAT-gal*-positive cells in the subpopulations of DRG precursors shows that only small fraction of the Neurog1 population express BAT-gal, in comparison to the other subpopulations. NT, neural tube; Dashed lines frame *in vivo* fate mapped cells, * indicates *P* <0.05; Scale bars: 25 μm. DRG, dorsal root ganglia; E, embryonic day.

Previous work from our lab also using *Wnt1-Cre*-mediated ablation of β-catenin, demonstrated the requirement of β-catenin for the expression of Neurog2 and Neurog1. However, it was unclear whether the virtual absence of Neurog1 expression in the mutant reflected a role of β-catenin in Wnt signaling or in mediating cadherin-dependent cellular adhesion [18]. Therefore, we analyzed protein expression of Neurog2 and Neurog1 in recombined cells expressing β-gal from the *R26R* lineage tracer on transverse sections of our β-catenin mutant models at E9 and E10.5, respectively. A significant loss of Neurog2 expression was shown by immunohistochemistry in both mutant embryos at E9 (Figure 3B-D, O), indicating that expression of Neurog2 is dependent on TCF/Lef mediated transcription. These results concur with previous findings that specified Neurog2 as a Wnt target [35].

Expression of Neurog1 was lost in *βcat-Null R26R* embryos at E10.5, as expected [18]. Interestingly, however, Neurog1 expression was retained in *βcat-Sig* embryos (Figure 3G-H, O). Considering that the mutated β-catenin-dm protein (expressed from *Ctnnb1*^*dm*^) lacks the ability to attract transcriptional co-activators to TCF/Lef transcription factors [15], the expression of Neurog1 in *βcat-Sig R26R* animals suggests that transcription of Neurog1 is not connected with the function of β-catenin as an activator of TCF/Lef-dependent transcription.

These data were supported by the results of co-stainings for early subtypes of DRG precursors and β-gal in control animals carrying the *BAT-gal* Wnt-reporter. Quantifications revealed that at least 40% of Neurog2- and Sox10-positive cells were positive for β-galactosidase at E9 (Figure 3J-K, P) and that at E10.5 the percentage of the Sox10-positive population expressing β-gal was still about 30% (Figure 3N, P). However, fewer than 15% of the Neurog1-positive cells were also positive for β-galactosidase (Figure 3M, P). These findings revealed that β-catenin-TCF/Lef transactivation is rather active in the Sox10-positive DRG progenitors and the first sensory precursor wave expressing Neurog2. In contrast, Wnt-induced TCF/Lef-driven transcriptional activation is apparently less prominent during the second wave of sensory neurogenesis characterized by Neurog1 expression.

**Figure 4:**
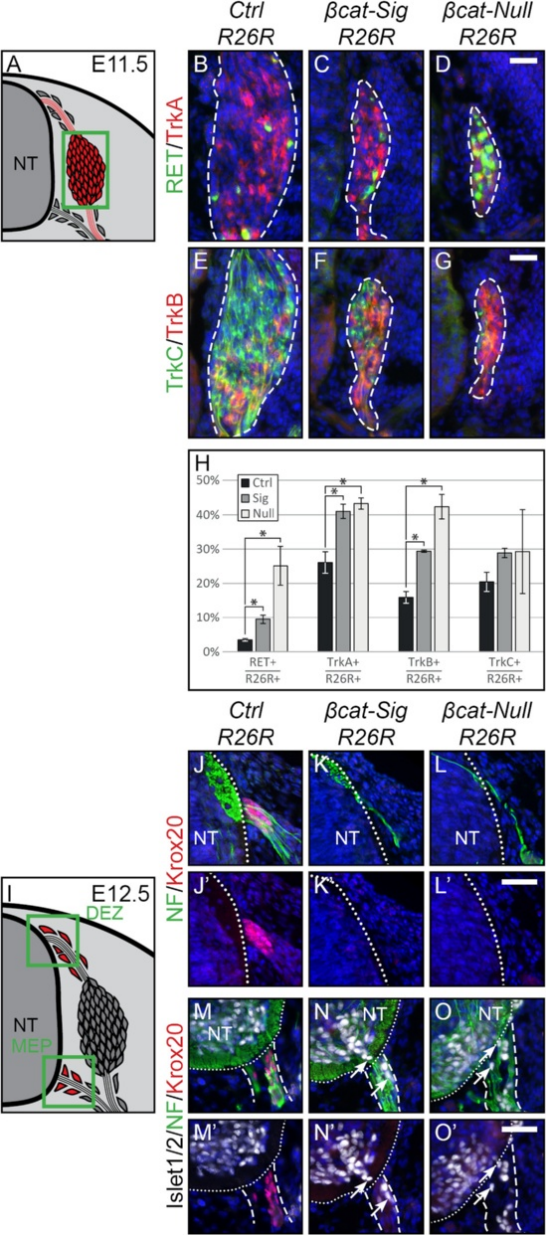
Late subtype specification is altered and boundary cap cell identity is lost in mutant animals. **(A)** Illustration of a transverse section at E11.5 displaying cells, which contribute to the DRG in red. Green box represents caption area for subfigures B-G. **(B-G)** Triple immunohistochemistry at E11.5 for β-gal expressed by the *R26R* reporter and for markers for subtype specification of sensory neurons: tyrosine receptor kinases RET and TrkA, TrkB and TrkC. Dashed lines surround the border of *R26R*-positive cells contributing to the DRG. **(H)** Quantification of late sensory subtypes per DRG displays an altered distribution of sensory neuron subtypes in mutant animals. **(I)** Illustration of a transverse section at E12.5 displaying boundary cap cells in red. Green box represents caption area for subfigures J-O, respectively. **(J-L)** Double immunohistochemistry for Krox20 as a marker for differentiated boundary cap cells and neurofilament (NF) at E12.5. **(M-O)** Triple immunohistochemistry for Krox20, NF and Islet1/2 as a marker for motor neurons at E12.5. **(J’,M’)** In control animals, sensory axons invading the dorsal entry zone of the neural tube and motor axons penetrating the prospective motor exit point of the neural tube are surrounded by boundary cap cells. **(K’,L’,N’,O’)** Dorsal as well as ventral Krox20 expression is lost in both mutant animals. Arrows show migrating motor neurons, which are exiting or have already exited the ventral neural tube due to the loss of functional boundary cap cells. NT, neural tube; DEZ, dorsal entry zone; MEP, motor exit point; Scale bars: 25 μm. DRG, dorsal root ganglia; E; embryonic day.

In addition to early markers of sensory neurogenesis, we also analyzed early subtype specification of the differentiating sensory neurons at E11.5, by staining for TrkA, TrkB, and TrkC, as well as a further Trk of sensory neurons RET. The relative contribution of the specific subtypes with the exception of TrkC varied between both mutant embryos to each other and the control (Figure 4B-G, H), reflecting subtype specification regulated by both Neurog1 and Neurog2.

The earliest marker for BCCs, representing the third wave of sensory neurogenesis, is the zinc finger transcription factor Krox20 (respectively, EGR2). Triple staining for Krox20, neurofilament and the transcription factors Islet1/2, which mark motor and sensory neurons, showed a loss of dorsal as well as ventral Krox20-expressing BCCs at E12.5 in both β-catenin mutant embryos (Figure 4, J-L). Krox20 has been implied to be a Wnt target [36]. Interestingly, Sox10-positive cells were observed at the dorsal entry zone and the motor exit point of both mutants at E12.5, suggesting that specification of BCCs rather than localization of their precursors was affected in β-catenin mutant embryos (Additional file 1: Figure S6D-I). Furthermore, emigration of motor neurons from the ventral neural tube was apparent in the ventral roots of the mutants (Figure 4, M-O). Such ectopic localization of motor neurons has been attributed to the loss of ventral BCCs or their secretion of constraining signals [37-39]. As stated before, no irregular apoptosis was detected in DRG progenitors including BCCs (data not shown). These data indicated that BCC precursors are present, but that the formation of functional BCCs is dependent on β-catenin as inducer of TCF/Lef transcription.

In summary, the expression of the transcription factors Neurog2 and Krox20 depends on β-catenin as co-transcription factor for TCF/Lef-mediated transcription. In contrast, Neurog1 expression involves a co-transcription-independent function of β-catenin, indicating that sensory neuronal subtypes in the DRG are specified by distinct mechanisms.

### Development of the dorsal root ganglia is independent of β- and α-catenin-encompassing cadherin adhesion junctions

As shown above, *βcat-Null* animals exhibited more severe phenotypes than *βcat-Sig* mutants, with first phenotypic variations manifested at E10.5 when the developing DRG coalesce laterally of the neural tube. The presence of N-cadherin and α-catenin at E12.5 (Additional file 1: Figure S4A-F) suggested that cadherin/β-catenin/α-catenin-mediated adhesion takes place in the DRG. Immunohistochemistry for β-catenin at the same stage confirmed the loss of β-catenin in the *βcat-Null* and only the presence of the mutated form of β-catenin in the *βcat-Sig* mutant (Additional file 1: Figure S4G-I). Since cadherin-mediated adhesion is preserved in *βcat-Sig* but not *βcat-Null* mutants, the differences between mutant embryos might possibly be a consequence of insufficient adhesion in *βcat-Null* embryos. To test this idea, we also analyzed *Wnt1-Cre Ctnna1*^*flox/flox*^ (*αcat-Adh*) mice, in which α-catenin is conditionally deleted in NCCs [40,41]. α-Catenin connects the actin-cytoskeleton with β-catenin bound to proteins of the cadherin family. Therefore, removing either α-catenin or β-catenin in the cadherin adhesion complex disrupts the connection of transmembrane-bound cadherins with the cytoskeleton, thus interfering with cell-cell adhesion. Accordingly, in both *βcat-Null* and *αcat-Adh* embryos, the epithelial integrity of the *Wnt1-Cre*-expressing dorsal neural tube was disturbed [15] (Additional file 1: Figure S5).

**Figure 5:**
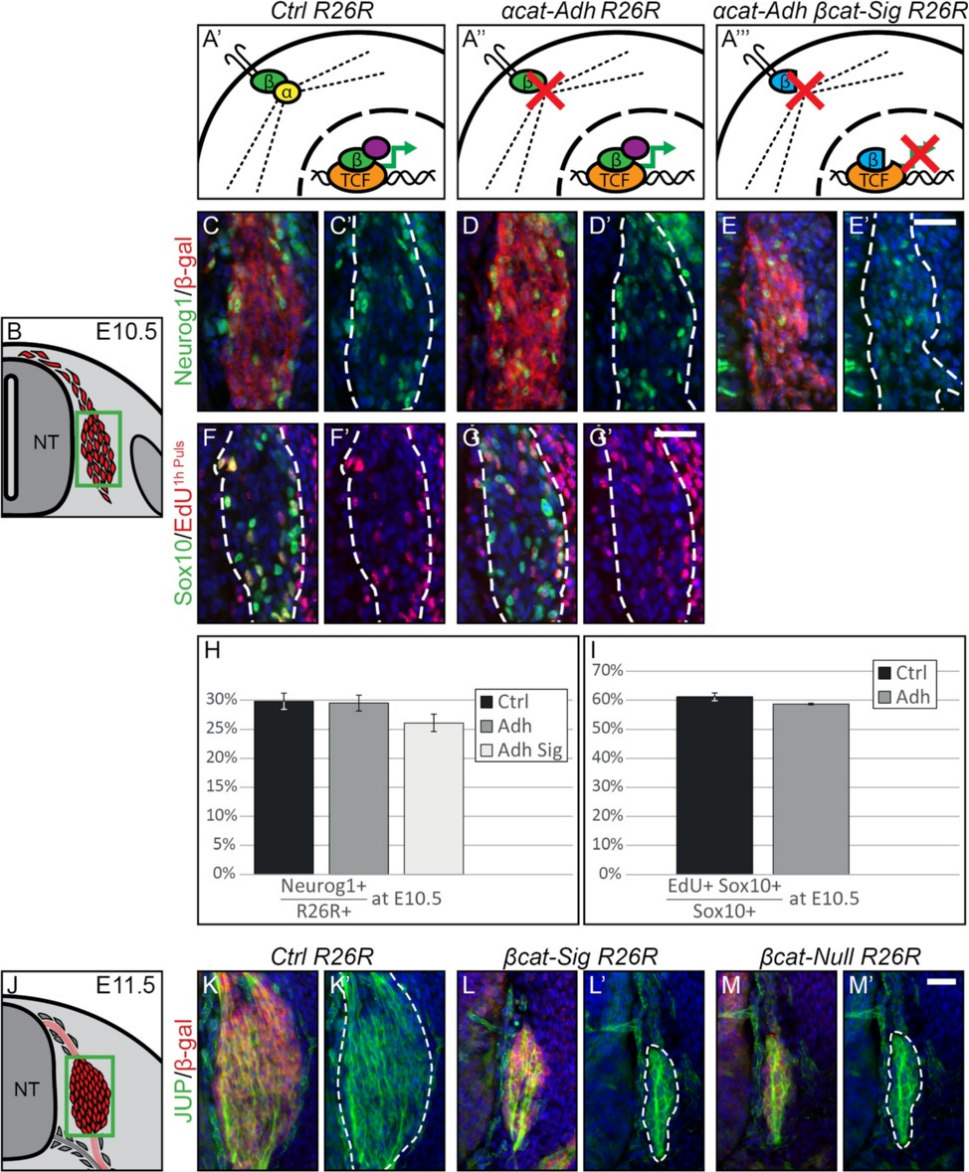
Development of the dorsal root ganglia does not depend on structural function of cadherin-mediated adherens junctions. **(A’-A”’)** Scheme representing functional properties of Cre-recombined cells in control and *αcat-Adh* and *αcat-Adh βcat-Sig* mutant animals. **(A’)** β-catenin (green) of control animals induces transcription (green arrow) by binding with TCF/Lef (orange) and recruiting co-transcription factors (purple). Furthermore, it links transmembrane cadherins via α-catenin (yellow) to the actin cytoskeleton (dotted line). **(A”)** The loss of α-catenin prevents binding of cadherin to the actin cytoskeleton. **(A”’)** In *αcat-Adh βcat-Sig* animals, the β-catenin-dm inhibits TCF/Lef-mediated transcription. Furthermore, the loss of α-catenin leads to an absence of cadherin-mediated adhesion. **(B)** Illustration of a transverse section at E10.5 displaying DRG progenitor cells in red. Green box represents caption area for subfigures C-G. **(C-E)** Double immunohistochemistry for Neurog1 and β-gal expressed by the *R26R* reporter. **(C’-E’)** Expression of Neurog1 is maintained in both mutant animals. **(F-G)** Immunofluorescent staining for Sox10 and detection of a one hour EdU pulse. **(F’-G’)** EdU incorporation of Sox10-positive DRG progenitor cells is preserved in *αcat-adh R26R* embryos. **(H)** Quantification of Neurog1-positive cells per *DRG* show no significant differences between control, *αcat-Adh R26R*, and *αcat-Adh βcat-Sig R26R* mutants. **(I)** Percentages of EdU-positive cells in the Sox10-positive population display no significant reduction of EdU incorporation in *αcat-Adh R26R* animals. **(J)** Illustration of a transverse section at E11.5 displaying DRG in red. Green box represents caption area for subfigures K-M. **(K-M)** Double immunohistochemistry for β-gal expressed from the *R26R* reporter and junction plakoglobin (JUP) shows the same expression density of JUP in β-catenin mutant animals as in control animals. **(K’- M’)**, corresponding single fluorescence channels of the sections shown in K, L, and M, respectively. Dashed lines frame *in vivo* fate-mapped cells. NT, neural tube; Scale bars: 25 μm. DRG, dorsal root ganglia; E, embryonic day; EdU, 5-ethynyl-2′-deoxyuridine.

Intriguingly, unlike in *βcat-Null R26R* embryos at E10.5 (Figure 3H), DRG progenitors of *αcat-Adh R26R* embryos maintained expression of Neurog1 (Figure 5D, H). Thus expression of Neurog1, indicative for the second wave of sensory neurogenesis, is not dependent on adhesion alone. Moreover, there is a possibility that initiation but not maintenance of Neurog1 expression could depend on multiple inputs such as TCF/Lef transcription as well as down-stream factors of stabilized adhesion in the coalescing DRG. To exclude this possibility, we generated *Wnt1-Cre Ctnna1*^*flox/flox*^*Ctnnb1*^*flox/DM*^ (*αcat-Adh βcat-Sig*) double mutant embryos, which mimics *βcat-Null* embryos with respect to loss of adhesion and TCF/Lef transcription. If expression of Neurog1 depends on additive effects of these two functions, *αcat-Adh βcat-Sig* double mutants should display the same phenotype as the *βcat-Null*. However, transverse sections through DRG of E10.5 *αcat-Adh βcat-Sig R26R* embryos revealed expression of Neurog1 comparable to control animals (Figure 5C-E, H). Likewise, cell cycle progression as assessed by incorporation of EdU in Sox10-positive cells at E10.5 was normal in *αcat-Adh R26R* mutants (Figure 5F-G, I). Furthermore, DRG size was not affected in *αcat-Adh R26R* embryos at E12.5, unlike in *βcat-Sig* mice (data not shown).

These data suggest that the DRG does not depend on the cadherin adhesion complex for proper formation. To further assess this assumption, we stained control animals at E9, E10.5, and E11.5 for tight junctions and desmosomes, other mediators of cell adhesion. Tight junctions were undetectable in DRG progenitors, as no expression for ZO1 was found (data not shown). In contrast, the expression of the essential desmosome component junction plakoglobin (JUP) was absent in migrating NCCs at E9, but became evident from E10.5 onwards and was readily detectable in DRG progenitors by E11.5 (Figure 5K). However, neither proximal membrane localization nor protein presence of JUP was altered in *βcat-Sig R26R* or *βcat-Null R26R* embryos at E11.5 (Figure 5L-M). Thus, loss of β-catenin has no effect on development or sustainability of cell-cell adhesion in DRG progenitors, possibly because adhesion in the developing DRG is sufficiently sustained by β- and α-catenin-independent desmosomes, rather than by cadherin/β-catenin/α-catenin-mediated adherens junctions.

In sum, our data indicate that all deviations of phenotypes of DRG development between *βcat-Null* and *βcat-Sig* mutants are not a result of intact cadherin-mediated adhesion in the *βcat-Sig* animals. Rather, Neurog1 expression, as well as proliferation of postmigratory Sox10-positive progenitors in developing DRG, requires a function of β-catenin independent of its role in attracting transcriptional co-activators to activate TCF/Lef transcription or to sustain cadherin-based adhesion.

### Expression of TCF/Lef transcription factors during DRG development

**Figure 6:**
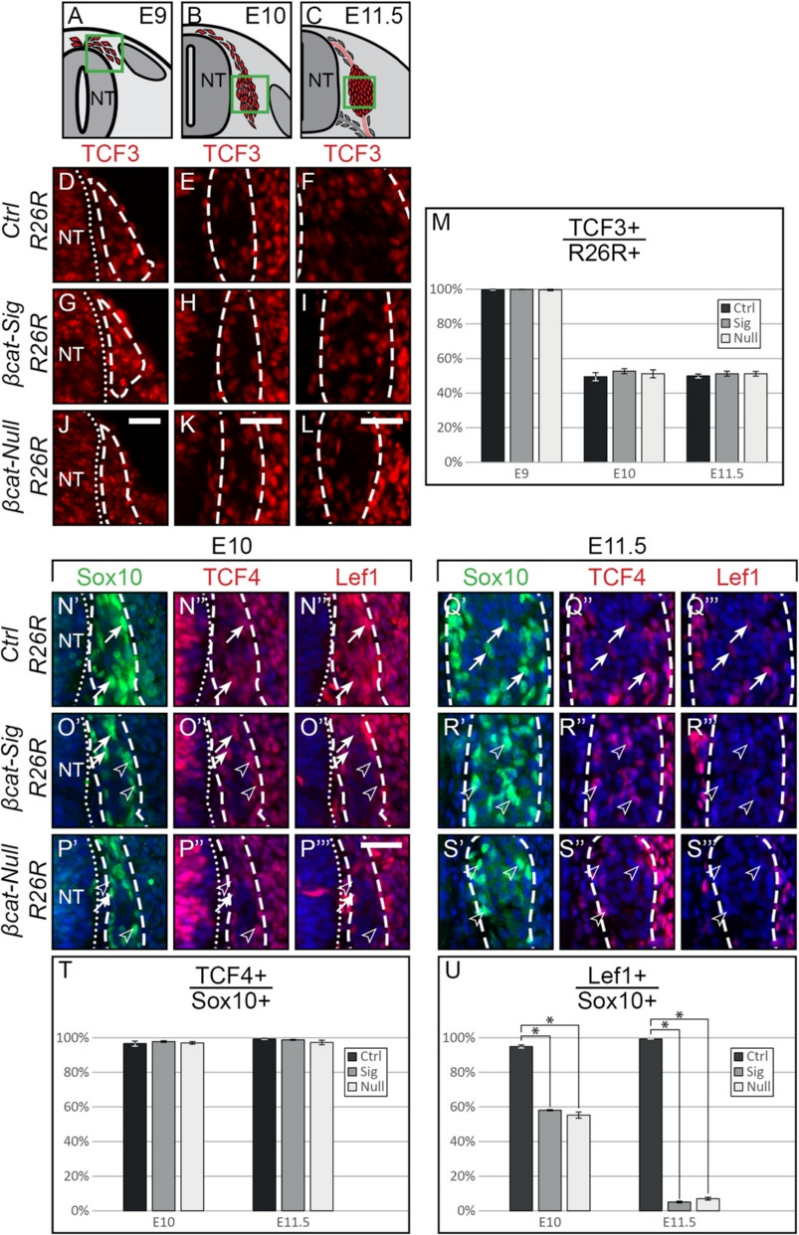
Expression of TCF3, TCF4, and Lef1 in the developing DRG. **(A-C)** Illustration of a transverse section at E9, E10 and E11.5, respectively, displaying cells which contribute to the DRG in red. Green box represents caption area for subfigures D-L, and N-S respectively. **(D-L)** Immunohistochemistry for TCF3 shows decreased TCF3 expression in control and mutant animals at E10 and E11.5 as compared to the situation at E9, as verified by quantification **(M)**. Dashed lines frame *in vivo* fate-mapped cells. **(N-S)** Triple immunohistochemistry for Sox10, TCF4 and Lef1. (**N’-S’, N”-S”, N”’-S”’**), single fluorescence channels of the same sections. **(T)** Quantification of TCF4 presence in Sox10-positive cells shows continuous expression in DRG cells and no difference between any of the three animals at both E10 and E11.5. **(U)** Quantification of Sox10- and Lef1-double positive cells shows reduced numbers in both mutants at E10 and a complete loss at E11.5. Dashed lines frame the outer border of cells which express Sox10 and thus roughly encompass the DRG area. Arrows point to cells expressing Sox10, TCF4 and Lef1. Arrow heads point to Sox10- and TCF4-positive, Lef1-negative cells. NT, neural tube; Scale bars: 25 μm. DRG, dorsal root ganglia; E, embryonic day.

Previously, it has been shown that TCF family factors, such as TCF3 and TCF4, can also function as repressors, independent from Wnt-induced transcriptional activation [5-12]. In this case, target genes are de-repressed by binding of β-catenin to the repressing TCFs, followed by release of the complex from the DNA binding sites. It is conceivable that β-catenin expressed from the *Ctnnb1*^*d*m^ allele preserves this function, in addition to rescuing cadherin-mediated adhesion. To address whether expression of TCFs during DRG development is compatible with a role of β-catenin in de-repression, we first stained embryonic sections for TCF3 at stages E9, E10 and E11.5 in control embryos. TCF3 expression was present in virtually all migratory NCCs at the early stage of E9. Subsequently, however, TCF3 expression decreased, and only about 50% of all DRG cells expressed TCF3 at E10 and E11.5 (Figure 6D-F, M). De-repression upon binding of β-catenin leads to partial TCF3 degradation, which is particularly evident in quantitative assays not applicable to β-catenin-mutant DRG [9]. Using immunohistochemistry, TCF3 expression levels appeared to be generally low in the forming DRG and not altered in β-catenin-mutant embryos as compared to the control (Figure 6G-L, M).

At E10 and E11.5 triple immunohistochemistry for Sox10, TCF4, and Lef1 revealed that TCF4 and Lef1 were consistently expressed within the Sox10 population of the forming DRG in control animals (Figure 6N, Q). De-repression mediated by TCF4 has not been shown to lead to its degradation. Accordingly, expression of TCF4 in Sox10-positive cells did not change in either mutant at any stage (Figure 6N”-S”, T). In contrast, the percentage of Lef1-positive cells was decreased by more than 40% at E10 and was below 10% at E11.5 in both mutants (Figure 6N”’-S”’, U). These data are in agreement with the known regulation of Lef1 by a positive feedback loop of Wnt signaling [42,43]. As of E12.5, Lef1 was no longer expressed in DRG cells of wild type animals with the exception of the BCCs, which were, however, devoid of Lef1 expression in *βcat-Sig* and *βcat-Null* embryos (Additional file 1: Figure S6).

In sum, the decrease of Lef1 expression from E10 to E11.5 in both mutants indicated that TCF/Lef transactivation is not essential for the induction of Lef1 expression, but necessary for its maintenance. Interestingly, Lef1 expression diminished in the DRG as of E12.5 in control animals, consistent with our finding that proliferation of DRG cells at later developmental stages is apparently not controlled by Wnt/β-catenin-induced transcriptional activation (Figure 2). Importantly, however, the expression of TCF3 and, in particular, TCF4 is compatible with an implication of β-catenin-dm in de-repression of TCF target genes.

### The sensory lineage of neural crest cells depends on secreted Wnt of non-neural crest origin

**Figure 7:**
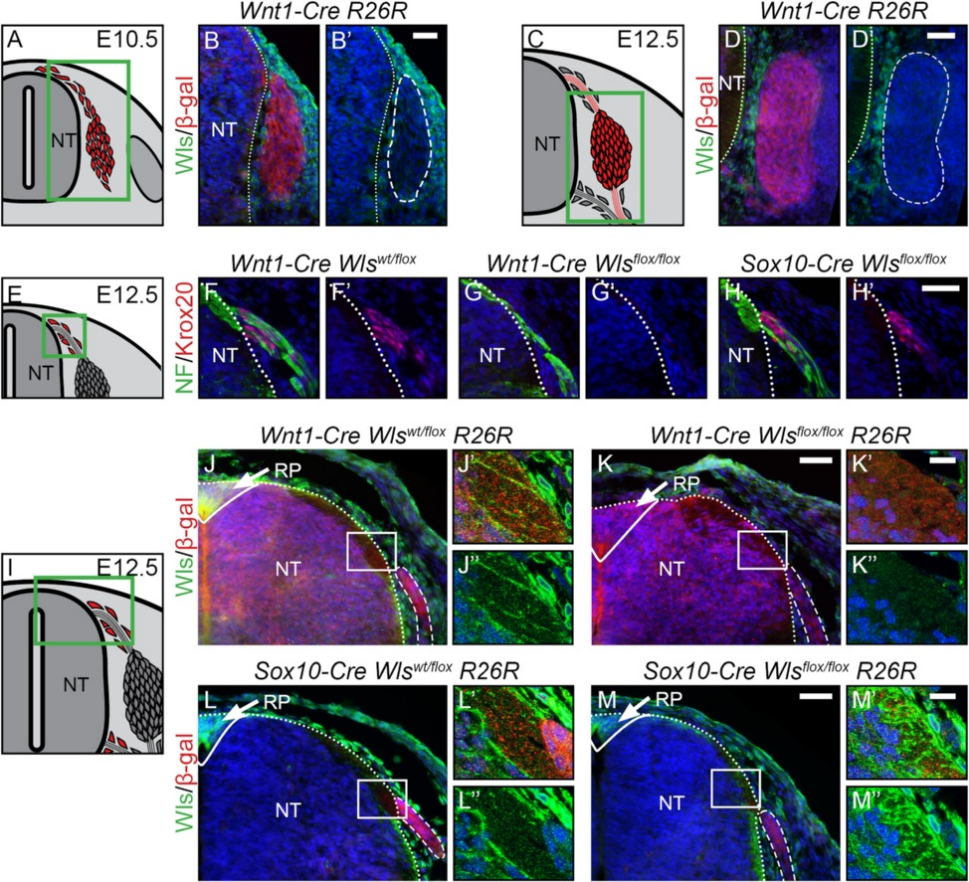
Development of boundary cap cells depends on neural tube-derived Wnt. **(A, C)** Illustration of a transverse section at E10.5 **(A)** and E12.5 **(C)** displaying cells contributing to the DRG in red. Green box represents caption area for subfigures B and D. respectively. **(B, D)** Double immunohistochemistry for Wls and β-gal expressed in *Wnt1-Cre R26R* mice shows that mesenchymal tissue and not cells populating the DRG express Wls. **(B’, D’)** Corresponding single fluorescence channels of the same sections. Dashed lines frame lineage-traced cells. **(E, I)** Illustration of a transverse section at E12.5 displaying BCCs in red. Green box represents caption area for subfigures F-H and J-M, respectively. **(F-H)** Double immunohistochemistry for Krox20 and neurofilament (NF). **(F’-H’)** Corresponding single fluorescence channels of the same sections. **(G’)** Dorsal BCCs are lost upon conditional knock-out of *Wls* using *Wnt1-Cre*. **(H’)** However, BCCs are rescued in conditional knockout of *Wls* using *Sox10-Cre*. **(J-M)** Double immunohistochemistry for Wls and β-gal expressed by the Cre reporter line *R26R*. J’-M’ and J”-M” are higher magnifications of the boxed areas in J-M, respectively. Dashed lines frame sensory axons**. (J-J”, L-L”)** Wls is expressed at the site where sensory axons enter the dorsal neural tube at the dorsal entry zone and strongly expressed in the roofplate (arrow, J, L) of control animals. **(K-K”)** Expression of Wls is lost in dorsal neural tube upon conditional knock-out of *Wls* using *Wnt1-Cre*, as evident in the roofplate (arrow, K) and the dorsal entry zone **(K’,K”)**. **(M-M”)** Conditional ablation of *Wls* using *Sox10-Cre* does not affect expression of Wls in the dorsal neural tube in either the roofplate (arrow, M) or the dorsal entry zone **(M’,M”)**. NT, neural tube; Dotted line show NT border; RP, roofplate; Scale bars: 25 μm; 10 μm in magnifications **J’-M’**. BCCs, boundary cap cells; DRG, dorsal root ganglia; E, embryonic day.

The availability of the multipass transmembrane protein Wntless (Wls, respectively Gpr177) is vital for the secretion of all Wnt proteins [44-46], even though expression of Wnt proteins is controlled by various mechanisms depending on the developing tissue. Interestingly, however, immunohistochemistry for Wls displayed no expression in DRG progenitors at E10.5 or E12.5, but rather in the surrounding mesenchyme and the neural tube (Figure 7B, D). DRG progenitors where visualized by co-staining for β-gal expressed from the R26R reporter allele recombined by *Wnt1-Cre*. To further assess that Wnt proteins necessary for sensory neurogenesis are not secreted by the DRG progenitors themselves, we utilized a mouse model, in which Wls was conditionally deleted (*Wls*^*flox*^) (Additional file 1: Figure S7) using *Wnt1-Cre*. Efficient deletion of Wls was determined by immunohistochemistry in *Wnt1-Cre*-recombined tissues, which express Wls, such as the roof plate (Figure 7J, K). Interestingly, no phenotype could be observed in early DRG progenitors, and the DRG of E12.5 mutant embryos were of normal size (data not shown). Surprisingly, however, identical to the phenotype observed in our β-catenin mutants, differentiated dorsal BCCs were missing upon ablation of Wls with *Wnt1-Cre*, as assessed by Krox20 staining (Figure 7F-G). One possible explanation for this phenotype is that Wnts necessary for dorsal BCC formation are secreted from the dorsal neural tube, which is in close vicinity to BCCs and also recombined in the *Wnt1-Cre* mouse line. Indeed, confocal images of a double staining for Wls and β-gal showed that in *Wnt1-Cre R26R* control animals, Wls was expressed at the site of the dorsal entry point of sensory neurons in the dorsal neural tube (Figure 7J’-J”). *Wnt1-Cre Wls*^*flox/flox*^ animals displayed a loss of Wls expression at the dorsal entry point (Figure 7K’-K”). To address the implication of neural tube-derived Wnt in BCC formation, we used a *Sox10-Cre* mouse line that confers Cre-mediated recombination in NCCs only after their emigration from the neural tube [19,47]. Use of this mouse line allowed the preservation of Wls expression in the dorsal neural tube and thus its capacity to secret Wnt, despite conditional deletion of Wls in NCCs (Figure 7L-M). Of note, Krox20 expression was rescued using the *Sox10-Cre Wls*^*flox/flox*^ mouse model in comparison with the *Wnt1-Cre Wls*^*flox/flox*^ model (Figure 7H).

Taken together, our data and previous reports [18-24] indicate that autocrine Wnt secretion is necessary in a short-time window for NCC delamination to occur. After delamination, however, the Wnt necessary for proper DRG development is not secreted by the DRG progenitors themselves. In particular, the development of dorsal BCCs depends on Wnt secreted from the dorsal neural tube.

## Discussion

β-catenin is a multifaceted regulatory protein that apart from mediating β-catenin-dependent cellular adhesion elicits various transcriptional responses in a context-specific manner [13]. These signaling responses often involve activation of TCF/Lef transcription factors, but can also be independent of TCF/Lef activation. In the present study, we use sensory neurogenesis and ganglia formation as a model system to demonstrate how distinct facets of β-catenin function to control cell proliferation and fate specification in a cell type- and stage-specific manner. Unexpectedly, the adhesion function of β-catenin is dispensable for sensory neurogenesis and the formation of DRG, as revealed by the comparison of conditional *βcat-Null*, *βcat-Sig*, and *αcat-Adh* embryos. β-catenin signaling, on the other hand, is essential for proper DRG development. However, β-catenin exploits both TCF activation-dependent and -independent signaling to regulate proliferation at different developmental stages as well as distinct cell fates associated with consecutive waves of sensory neurogenesis.

### Sensory ganglia formation does not require β-catenin/α-catenin-mediated cellular adhesion

Cadherin adherens junctions associate transmembrane cadherin proteins with the actin cytoskeleton via a β-catenin - α-catenin complex. Cellular adhesion mediated by this process might conceivably be important for DRG formation, because DRG size and cellular composition were severely affected in conditional *βcat-Null* but much less so in *βcat-Sig* mutant animals. In the dorsal neural tube used as a control tissue, the deletion of either β-catenin or α-catenin resulted in loss of cell-cell adhesion and, thus, in the disruption of the epithelial integrity. In contrast, deletion of α-catenin did not affect DRG formation or growth, unlike loss of β-catenin in *βcat-Null* embryos. Crossing *αcat-Adh* with *βcat-Sig* animals also did not produce the phenotype of *βcat-Null* mutant DRG, excluding any compensatory mechanisms between TCF/Lef transcription and down-stream factors of stabilized adhesion. Additionally, we showed that DRG cells and their projecting axons commence to express plakoglobin as the DRG coalesces and maintain this expression with further development. Taken together, these findings substantiate that cadherin adhesion junctions are negligible for sensory neurogenesis and that cell-cell adhesion in the developing DRG might be mediated via desmosomes. Moreover, the differences in DRG formation observed in *βcat-Sig* versus *βcat-Null* embryos can apparently not be attributed to an adhesion phenotype. This, in turn, suggests that the mutated form of β-catenin in *βcat-Sig* mice not only maintains the ability to preserve cadherin-mediated adhesion, but also rescues a function of β-catenin independent of its function as an activator of TCF/Lef transcription.

### Different functions of β-catenin regulate stage-specific proliferation of dorsal root ganglia progenitor cells

**Figure 8:**
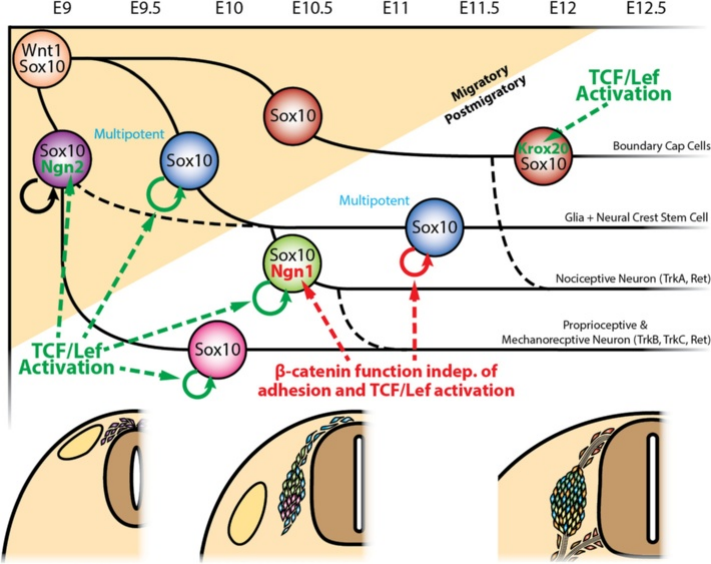
Development of the dorsal root ganglia depends on varying functions of β-catenin during sensory neurogenesis. Simplified scheme illustrating the genetic cascades that control sensory neurogenesis [26] and their dependency on functions of β-catenin. The expression of Neurog2 specific for the first wave of neurogenesis and expression of Krox20 specific for the third wave depends on β-catenin to induce TCF/Lef-mediated transcription (green dashed line arrows). Furthermore, proliferation for late migratory and early postmigratory cells depends on one or more downstream factors of TCF/Lef transactivation (green dashed line arrows). Expression of Neurog1 as well as proliferation of postmigratory cells from E11 onwards depends on a function of β-catenin independent of its role to induce TCF/Lef transcription or to constitute cadherin-mediated cell-cell adhesion. E, embryonic day.

NCCs at different stages of development are subject to distinct mechanisms of proliferation control [48]. This most probably reflects the changing environment NCCs encounter in the tissue they migrate through and the niches they reside in post migration. At early stages of migration, NCC proliferation was neither affected in *βcat-Sig* nor in *βcat-Null* embryos, consistent with earlier reports that canonical Wnt signaling does not regulate proliferation of early NCCs [18,21]. However, at E10.5, post-migratory DRG progenitors of *βcat-Sig* and *βcat-Null* mutant embryos failed to incorporate EdU after a one hour pulse even though they expressed KI67. These results indicate that DRG progenitors in both mutant animals are stuck in cell cycle, most probably in G1 phase. Wnt signaling has been shown to regulate cell proliferation by promoting G1 progression [31]. Moreover, we found Lef1 to be expressed at E10 and E11.5 within the Sox10 population of control animals, while its expression was lost at E12.5 within the DRG. Furthermore, reduction of Lef1 in both *βcat-Sig* and *βcat-Null* mutants was observed at E10 and E11.5. Therefore, at a stage during development when NCCs coalesce to form DRG, cell cycle progression of DRG progenitors is controlled by Wnt/β-catenin-dependent transcriptional activation, possibly mediated by Lef1 (Figure 8, green arrow). Intriguingly, at somewhat later stages of DRG development (E11.5 onwards), the DRG progenitors of the *βcat-Sig* mutants recovered their capacity to progress through the cell cycle, whereas the *βcat-Null* mutant cells were still incapable of doing so. Thus, the mutant form of β-catenin expressed in *βcat-Sig* animals is apparently able to rescue DRG progenitor proliferation at later developmental stages.

As described above, the differences in the phenotype between *βcat-Sig* and *βcat-Null* mutant DRG are not due to changes in cadherin-mediated adhesion. We propose, therefore, that an alternative, adhesion- and TCF/Lef-transactivator-independent function of β-catenin regulates one or multiple factors responsible for the induction of a second wave of proliferation in DRG (Figure 8A, red arrow). The expression of the known repressors TCF3 and, in particular, TCF4 [5-12] in the developing DRG allows us to speculate, that this function likely involves the ability of β-catenin to de-repress TCF-bound target genes. In summary, NCC proliferation during migration and in specific NC target structures appears to be dynamically controlled by stage- and location-specific mechanisms that involve β-catenin-independent factors [48] as well as diverse β-catenin-dependent signaling pathways.

### Specification of the three waves of sensory neurogenesis depends on distinct β-catenin signaling functions

Subtype specification of sensory neurons is driven by proneural transcription factors, which regulate the three sequential waves of neurogenesis. The first wave is initiated in mice at around E9 and consists of migratory Sox10- and Neurog2-double positive cells fated to become mechano- or proprioceptive neurons expressing TrkB, TrkC and RET [26]. However, lineage tracing revealed that some Neurog2-positive cells also give rise to TrkA-expressing neurons [49]. The second wave of neurogenesis is characterized by the continuous expression of Sox10 throughout migration and thereafter in the coalescing DRG. These multipotent DRG progenitors divide at a very high rate [50] and then either give rise to a subpopulation of sensory neurons or glial cells, or remain DRG progenitors. The subgroup designated to become sensory neurons express Neurog1 post migration and bias cells to become nociceptive neurons expressing TrkA and Ret, but they can also give rise to mechano- and proprioceptive neurons [32]. The third and final wave of sensory neurogenesis refers to the generation of BCCs. These cells express Krox20 and produce peripheral glia along the sensory axons entering the dorsal entry zone and motor axons exiting the ventral neural tube at prospective motor exit points [34]. However, a subgroup of these cells loses their Krox20 expression, migrate into the developing DRG and give rise to nociceptive neurons [34].

In both *βcat-Sig* and *βcat-Null* animals, expression of Neurog2 and Krox20 was absent, indicating that these transcription factors are regulated by TCF/Lef-mediated transcription (Figure 8, green arrows). In the case of Krox20, which has been proposed to be a TCF/Lef transcriptional target [36], the responsible transcription factor likely is Lef1, which is strongly expressed within the BCCs. In contrast, Neurog1 was lost in *βcat-Null* mutants, but was still expressed in *βcat-Sig* animals. The expression of Neurog1 was not dependent on the capacity of the *βcat-Sig* mutant to preserve cadherin-mediated adhesion, as expression of Neurog1 was lost neither in *αcat-Adh* nor in *αcat-Adh βcat-Sig* embryos. Promoter binding studies performed in CNS progenitor cells have previously indicated that β-catenin can regulate Neurog1 gene expression [51,52]. Thus, given the normal expression of Neurog1 in *βcat-Sig* DRG, a function of β-catenin independent of its role in adhesion and TCF/Lef-induced transcriptional activation is responsible for Neurog1 expression. In support of this, *BAT-gal* reporter activity was low in Neurog1-expressing cells, in contrast to Neurog2-positive cells. Although this remains to be proven, Neurog1 is likely a target gene of TCF3/4-mediated repression released upon binding of β-catenin.

Collectively, these findings indicate that the proneural transcription factors specific for each of the three waves of sensory neurogenesis depend on β-catenin independent of its role in the cadherin adhesion complex. Importantly, the expression of Neurog2 and Krox20 are dependent on the ability of β-catenin to attract transcriptional co-activators to initiate TCF/Lef transcription, whereas Neurog1 expression does not require β-catenin to function as a TCF-transactivator (Figure 8, red arrows).

Our data show that both stage-specific proliferation and progressive fate determination in DRG involve β-catenin functions. The successive waves of sensory neurogenesis could be regulated by spatiotemporal Wnt ligand expression in the DRG itself or by changing Wnt expression in the tissues adjacent to the growing DRG. In agreement with previous studies [53,54], *Wnt1-Cre*-mediated conditional deletion of *Wls* – which prevents secretion of any Wnts in NCCs and their derivatives – did not result in overt DRG malformation. Previous work has shown that non-conditional double knock-out of Wnt1 and Wnt3a, the mayor known Wnts expressed in the dorsal neural tube and premigratory NCCs, leads to major deficiencies in NCC derivatives, including the DRG [55,56]. The *Wnt1-Cre Wls*^*flox/flox*^ might not mimic this phenotype, because deletion of *Wls* occurs only after activation of the *Wnt1*-promotor. Our findings show that DRG progenitors are not subject to autocrine Wnt signaling after delamination. Rather, they depend on Wnt secretion from the surrounding tissue. In particular, BCCs emerge in close proximity to the neural tube, which is known to express various Wnt ligands [57]. Indeed, a thorough comparison between *Wnt1-Cre Wls*^*flox/flox*^ embryos (in which secretion of Wnts is lost in the dorsal neural tube and in NCCs) and *Sox10-Cre Wls*^*flox/flox*^ animals (in which secretion of Wnts is exclusively depleted from NCCs; [19]), revealed that Wnts derived from the neural tube promote the generation of BCCs, while Neurog2- and Neurog1-dependent sensory lineages require other Wnt sources, most likely provided by the mesenchyme adjacent to NCCs and the forming DRG. Thus, the different subpopulations of sensory progenitors not only depend on different β-catenin functions but also on distinct sources of Wnt ligands.

## Conclusions

### Adhesion and signaling independent functions of β-catenin during DRG development

Although canonical Wnt signaling has previously been implicated in neural crest development and, in particular, sensory neurogenesis [18,20,21,55], the dual function of β-catenin in mediating cell-cell adhesion and Wnt signaling made it difficult to study the actual contribution of this signaling pathway to peripheral ganglia formation and cell type specification. Therefore, we designed an animal model, which allowed us to dissect adhesion and signaling roles of β-catenin *in vivo* [15]. To definitely attribute phenotypic changes to defective adhesion, we also performed an analysis of *β-catenin*-deficient DRG. Surprisingly, however, our study reveals that several processes underlying DRG formation do neither depend on a structural role of β-catenin in adherens junctions nor solely to attract transcriptional co-activators for initiating TCF/Lef mediated transcription. Recently it has been shown that some TCFs also act as repressors for other modes of transcription [5,7-10,12,58]. The binding of β-catenin to these TCFs displaces the co-repressors and de-represses the TCF-target genes, allowing transcription by other transcription complexes. Although for technical reasons we were unable to address this hypothesis directly, it is conceivable that the mutated form of β-catenin present in *βcat-Sig* embryos is capable of maintaining the capacity to de-repress the TCF/Lef Groucho repression complexes, as its TCF/Lef-binding domain is preserved. This theory is supported by the fact that Neurog1 is expressed in the DRG progenitors of *βcat-Sig* animals, even though consensus sequences for TCF binding are located close to the transcription start site of the mouse Neurog1 gene [51] and β-catenin is directly involved in regulation of Neurog1 [52]. In any case, our findings demonstrate that the double mutated form of β-catenin in cells of *βcat-Sig* mice, although defective in TCF/Lef activation, preserves a specific signaling function of β-catenin, next to its ability to maintain proper formation of the cadherin adhesion complex. This made it possible to demonstrate that distinct β-catenin signaling functions regulate DRG size and cellular composition in a spatiotemporal manner. The interaction partners and targets mediating these stage-specific functions remain to be identified.

## Methods

### Animals and genotyping

Mouse experiments were performed in accordance with Swiss guidelines and approved by the Veterinarian Office of the Kanton of Zürich, Switzerland. The Cre-loxP system was used to conditionally knockout various genes. The generation of mutant mice has been reported previously: *Wnt1-Cre* mice [59], *Sox10-Cre* mice [47], *Ctnnb1*^*tm2Kem*^ (*Ctnnb1*^*flox*^) mice [25], *Ctnnb1*^*dm*^ mice [15], and *Ctnna1*^*tm1Efu*^ (*Ctnna1*^*flox*^) mice [41]. *In vivo* fate mapping was performed utilizing either the *ROSA26* reporter expressing β-galactosidase (*R26R*) [27] or the *ROSA26* reporter expressing *EGFP* [29] mouse lines. Embryos carrying one allele for lineage tracing are indicated as *R26R* or *EGFP*. To monitor Wnt/β-catenin transcription, animals were crossed with the *BAT-gal* reporter line [28].

To study the role and importance of Wls in the mouse, a conditional allele of *Wls* (*Wls*^*flox*^) was generated (Ozgene, Bentley, Australia). The targeting approach was to flank the first exon with loxP sites. The first exon encodes the transcriptional start and the complete signal sequence. Excision of the ‘floxed’ region, therefore, leads to a *Wls* null-allele. Bl6 was used as the background strain. The targeting vector was generated by assembling the 5’ and 3’ homology arms with the loxP site flanked first exon and the FRT site flanked neomycin selection cassette (Additional file 1: Figure S7). Deletion of the FRT flanked neomycin resistance cassette was verified by PCR and subsequent sequencing of positive Bl6 ES cell clones. *Wls* conditional homozygous mice are viable and fertile and do not show any abnormalities. Deletion of the loxP flanked first exon to generate the *Wls* knockout allele was verified by PCR and sequencing.

Genotyping for the floxed *Wls* allele was performed by PCR with primers Wls-up (5'-CCCCCTTTCCCTCTCGGTTCC-3') and Wls-lo (5'- GGCGGCATGGAAGCCAAGGGC -3') and 34 cycles of 94°C for 30 seconds, 57°C for 30 seconds and 72°C for 1 minute, to amplify a wild type fragment of 267bp length and, respectively, a mutant fragment 353bp in length.

### X-Gal staining and immunohistochemistry

*LacZ* reporter gene expression was detected using X-Gal staining [18]. For immunohistochemistry, cryo-sections were fixed for 30 seconds in 4% formaldehyde at room temperature and treated with blocking buffer (1% BSA, 0.3% Triton X-100, in PBS) for 30 minutes. Primary antibodies were used as follows: chicken anti-β-galactosidase (1:2000; Abcam ab9361, Cambridge, UK), rabbit anti-GFP(1:500; Abcam ab290), goat anti-Sox10 (1:200; Santa Cruz sc-17342, Dallas, Texas, USA), mouse anti-Neurog2 (1:10; gift from D. J. Anderson, California Institute of Technology, Pasadena, CA, USA), anti-Neurog1 (1:200; Santa Cruz sc-19231), goat anti-RET (1:500; Fitzgerald 70R-RG002X, Acton, Massachusetts, USA) rabbit anti-TrkA (1:500; gift from L.F. Reichardt, University of California, San Francisco, CA, USA), rabbit anti-TrkB (1:100; Cell signaling 4607, Danvers, Massachusetts, USA), goat anti-TrkC (1:50; R&D system Inc. AF1404, Minneapolis, Minnesota, USA), mouse anti-NF (1:250; Invitrogen 13-0700, Carlsbad, California, USA), rabbit anti-Krox20 (1:4000 gift from D. Meijer, Erasmus University Medical Center, Rotterdam, NL), mouse anti-JUP (1:200, BD Transduction laboratories 610253, Franklin Lakes, New Jersey, USA), rabbit anti-Wls(1:1000 Seven Hills Bioreagents, Cincinnati, Ohio, USA WLAB-177), rabbit anti-Ki67 (1:200, Abcam ab15580),mouse anti-β-catenin N-terminus (1:200; Enzo Life Sceince ALX-804-060-C100, Farmingdale, New York, USA), rabbit anti-β-catenin C-terminus (1:200; Sigma c2206, Buchs, St. Gallen, Switzerland), rabbit anti-cCasp3 (1:200; Cell Signaling 9661), mouse anti-Islet1/2 (1:200; Hybridoma 40.2D6, Iowa City, Iowa, USA), goat anti-TCF3 (1:200; Santa Cruz sc-8635) mouse anti-TCF4 (1:200; gift from Basler Lab; University of Zurich, Zurich, Switzerland), rabbit anti-Lef1 (1:500; Cell Signaling C12A5), rabbit anti-Brn3a (1:5000; gift from Turner Lab, Seattle Children’s Research Institute, Seattle, WA 98101, USA) rabbit anti-Ncad (1:200; Takara M142, Otsu, Shiga, Japan) rabbit anti-αcat (1:200; Sigma C-2081) mouse anti-ZO1 (1:200; Zymed 33-9100, Carlsbad, California, USA). Secondary antibodies were from Jackson Immuno Research, West Grove, Pennsylvania, USA or Invitrogen. Tyramide Signal Amplification (TSA) kit from PerkinElmer, Waltham, Massachusetts, USA was used for amplification of TCF3 and TCF4. EdU incorporation was stained using ‘click’ chemistry and was performed as described [60].

### Statistical analysis

All quantifications were performed on 12 μm transverse sections. Three control and three mutant embryos were quantified at each stage for each type of mutant. To determine rostral-caudal differences quantifications were performed in each embryo on levels of the upper and lower limbs as well as on an intermediate level between the two, collecting two to three sections for each level. As relative proportions were consistent on all three levels in all animal types, we averaged the output of eight sections per embryo. Statistical analyses were performed using the two-tailed unpaired Student’s t-test between control and mutant animals with Microsoft Excel. All results are shown as mean ± standard deviation. Pictures in figures were chosen from the intermediate sections between the upper and lower limbs.

## Additional file

Additional file 1:
**Supplemental Figures S1 to S7. Figure S1** (related to Figure 1): NCCs giving rise to the enteric nervous system, the sympathetic ganglia and the cardiac outflow tract are not subjected to canonical Wnt signaling as opposed to NCCs populating the pharyngeal arches. **Figure S2** (related to Figure 1): Proof of principle for the replacement of the endogenous β-catenin protein by the signaling mutant form in the DRG. **Figure S3** (related to Figure 2): The DRGs of *βcat-Sig* mutants do not recover normal DRG size at later stages. **Figure S4** (related to Figure 5): Presence of cadherin-adhesion complex components at E12.5. **Figure S5** (related to Figure 5): The dorsal neural tube upon loss of β-catenin or α-catenin displays identical loss of cell-cell adhesion. **Figure S6** (related to Figure 6): The absence of Lef1 expression at E12.5 in wild type DRGs and the loss of Lef1 expression in BCCs of mutant animals. **Figure S7** (related to Figure 7): Generation of the *Wls* ^*flox*^ allele.

## Abbreviations

BCCs: boundary cap cells
bHLH: basic helix-loop-helix
bp: base pair
BSA: bovine serum albumin
DRG: dorsal root ganglia
E: embryonic day
EdU: 5-ethynyl-2′-deoxyuridine
EGFP: enhanced green fluorescent protein
JUP: junction plakoglobin
NCCs: neural crest cells
PBS: phosphate-buffered saline
Trk: tyrosine receptor kinase
β-gal: β-galactosidase
