# Supplementary material for: Distinct adhesion-independent functions of β-catenin control stage-specific sensory neurogenesis and proliferation
Source: BMC Biol. 2015 Apr 11;13:24. doi: 10.1186/s12915-015-0134-4 (PMC4416270; doi:10.1186/s12915-015-0134-4)
Supplement: Additional file 1: — Supplemental Figures S1 to S7. Figure S1 (related to Figure 1): NCCs giving rise to the enteric nervous system, the sympathetic ganglia and the cardiac outflow tract are not subjected to canonical Wnt signaling as opposed to NCCs populating the pharyngeal arches. Figure S2 (related to Figure 1): Proof of principle for the replacement of the endogenous β-catenin protein by the signaling mutant form in the DRG. Figure S3 (related to Figure 2): The DRGs of βcat-Sig mutants do not recover normal DRG size at later stages. Figure S4 (related to Figure 5): Presence of cadherin-adhesion complex components at E12.5. Figure S5 (related to Figure 5): The dorsal neural tube upon loss of β-catenin or α-catenin displays identical loss of cell-cell adhesion. Figure S6 (related to Figure 6): The absence of Lef1 expression at E12.5 in wild type DRGs and the loss of Lef1 expression in BCCs of mutant animals. Figure S7 (related to Figure 7): Generation of the Wls flox allele. [file 12915_2015_134_MOESM1_ESM.pdf]

Additional file summary

Additional file 1: the file contains Figures S1 to S7

Figure S1 (related to Figure 1): NCCs giving rise to the enteric nervous system, the sympathetic ganglia and the cardiac outflow tract are not subjected to canonical Wnt signaling as opposed to NCCs populating the pharyngeal arches.

Figure S2 (related to Figure 1): Proof of principle for the replacement of the endogenous β-catenin protein by the signaling mutant form in the DRG.

Figure S3 (related to Figure 2): The DRGs of *βcat-Sig* mutants do not recover normal DRG size at later stages.

Figure S4 (related to Figure 5): Presence of cadherin-adhesion complex components at E12.5.

Figure S5 (related to Figure 5): The dorsal neural tube upon loss of β-catenin or α-catenin displays identical loss of cell-cell adhesion**.**

Figure S6 (related to Figure 6): The absence of Lef1 expression at E12.5 in wild type DRGs and the loss of Lef1 expression in BCCs of mutant animals.

Figure S7 (related to Figure 7): Generation of the $\textit{Wls}^{\textit{flox}}$ allele.

Additional files

Figure S1 NCCs giving rise to the enteric nervous system, the sympathetic ganglia and the cardiac outflow tract are not subjected to canonical Wnt signaling as opposed to NCCs populating the pharyngeal arches.

(A-D) Visualization of canonical Wnt activity using the *β-gal* expressing Wnt-reporter line *BAT-gal* in derivatives of NCCs, which were lineage traced using a *Wnt1-Cre* mouse line and *Cre*-induced EGFP. Double immunofluorescent staining β-gal and EGFP do not display co-localization of EGFP and β-gal in NCCs giving rise to progenitors of the enteric nervous system, the sympathetic ganglia, and the cardiac outflow tract at E10.5 **(A-C)**. However, EGFP-positive NCCs populating the pharyngeal arches express β-gal expressed by the *BAT-gal* transgene **(D)**.

Scale bars: 50 µm

Figure S2 Proof of principle for the replacement of the endogenous β-catenin protein by the signaling mutant form in the DRG.

(A) Illustration of a transverse section of an embryo at E10.5 displaying cells contributing to the DRG in red. Green box represents caption area for subfigures B-G.

(B-D) Double immunofluorescent staining for β-gal expressed by the *BAT-gal* transgene and the *Cre*-induced EGFP lineage tracer at E10.5. *Ctrl* animals show many β-gal-positive cells in the developing DRG **(B, B')**. Both the *βcat-Sig* and *βcat-Null* animals display an almost complete loss of β-gal expression in EGFP positive cells **(C-D')**.

(E'-G', E'''-G''', F''''-G'''') Single channels of triple staining for EGFP and the N- and C-terminus of β-catenin at E10.5. **(E''-G'')** Overlay of channels of staining for the N- and C-terminus of β-catenin. Only a staining for the N-terminus of β-catenin can be detected in DRG progenitors of *βcat-Sig* embryos expressing EGFP, demonstrating that wild type β-catenin has been replaced in these cells by the double mutated form with the truncated C-terminus **(F'-F'''')**. Furthermore, EGFP-positive cells of *βcat-Null* animals have completely lost expression of β-catenin **(G-G'''')**.

Scale bars: 25 µm

Figure S3 The DRGs of *βcat-Sig* mutants do not recover normal DRG size at later stages.

(A-B) Staining for neurofilament (NF) as well as **(C-D)** Brn3a shows a reduced size of the DRG in *βcat-Sig* embryos at E16.5

Scale bars: 50 μm

Figure S4 Presence of cadherin-adhesion complex components at E12.5.

(A-F) Immunofluorescent staining for N-cadherin (N-cad) or α-catenin (α-cat) reveal no alteration of their expression in either mutant.

(G-I) Overlay of channels of staining for the N- and C-terminus of β-catenin. Only a staining (red) for the N-terminus of β-catenin can be detected in DRG progenitors of *βcat-Sig* embryos, demonstrating that wild type β-catenin has been replaced in these cells by the double mutated form with the truncated C-terminus.

Dashed lines frame *R26R*-lineage traced cells, Scale bars: 50 µm

Figure S5 The dorsal neural tube upon loss of β-catenin or α-catenin displays identical loss of cell-cell adhesion**.**

(A'-A'''') Schematic of Cre-recombined cells and the functional output of the corresponding genotype. **(A')** β-catenin (green) of control animals induces transcription (green arrow) by binding with TCF/Lef (orange) in the nucleus and recruiting co-transcription factors (purple). Furthermore it links transmembrane cadherins via α-catenin (yellow) to the actin cytoskeleton (dotted line). **(A'')** The mutated β-catenin protein (blue) of *βcat-Sig* animals inhibits TCF/Lef-mediated transcription, but preserves cadherin-mediated adhesion. **(A''')** Cells of *βcat-Null* animals lose both TCF/Lef-mediated transcription and cadherin-mediated adhesion, as β-catenin is completely absent. **(A'''')** The loss of α-catenin in *αcat-Adh* animals prevents binding of cadherin to the actin cytoskeleton.

(B-E) Staining for ZO1 a marker for tight junctions on transverse sections of E12.5 embryos shows that a complete loss of β-catenin or α-catenin causes a disruption of the epithelial integrity of the dorsal neural tube and leads to cells entering the neural tube lumen **(arrows, D,E).** In contrast, epithelial integrity is preserved in *βcat-Sig* animals **(C).**

Scale bar: 100 µm

Figure S6 The absence of Lef1 expression at E12.5 in wild type DRGs and the loss of Lef1 expression in BCCs of mutant animals.

(A) Illustration of a transverse section at E12.5 displaying cells of the DRG in red. Green box represents caption area for subfigure B.

(B) Double-staining for Sox10 and Lef1 shows a loss of Lef1 expression in the entire DRG. Dashed lines frame lineage-traced cells.

(C) Illustration of a transverse section at E12.5 displaying boundary cap cells in red. Green box represents caption area for subfigures D-I, respectively.

(D-I) Double immunohistochemistry for Sox10 as a marker for boundary cap cell precursors and Lef1 at E12.5. **(D'-I')** In control animals, Sox10-positive cells in the vicinity of the dorsal entry zone and the motor exit point express Lef1. However, in both mutant animals, the same cell populations have lost Lef1 expression. **(K',L',N',O')**. Dashed lines border nerve bundles of the dorsal entry zone and the motor exit point, respectively.

NT, neural tube; DEZ, dorsal entry zone; MEP, motor exit point; Scale bars: 25 µm

Figure S7 Generation of *Wls*^{flox} allele.

For the generation of *Wls*^{flox} animals, the targeting vector was generated by assembling the 5' and 3' homology arms with the first exon flanked by loxP sites and the neomycin selection cassette flanked by FRT sites. Deletion of the FRT-flanked neomycin resistance cassette was verified by PCR and subsequent sequencing of positive Bl6 ES cell clones.

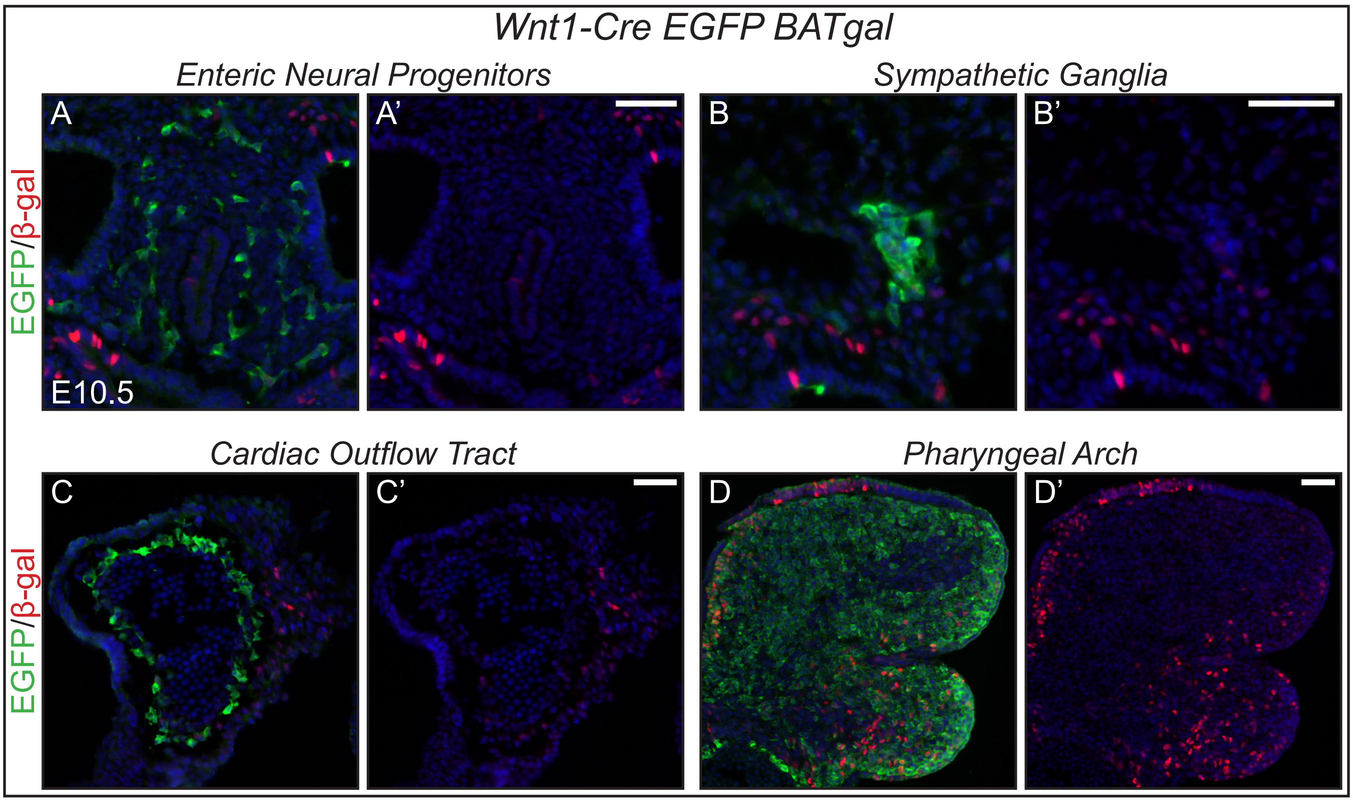

Additional file 1: Figure S1

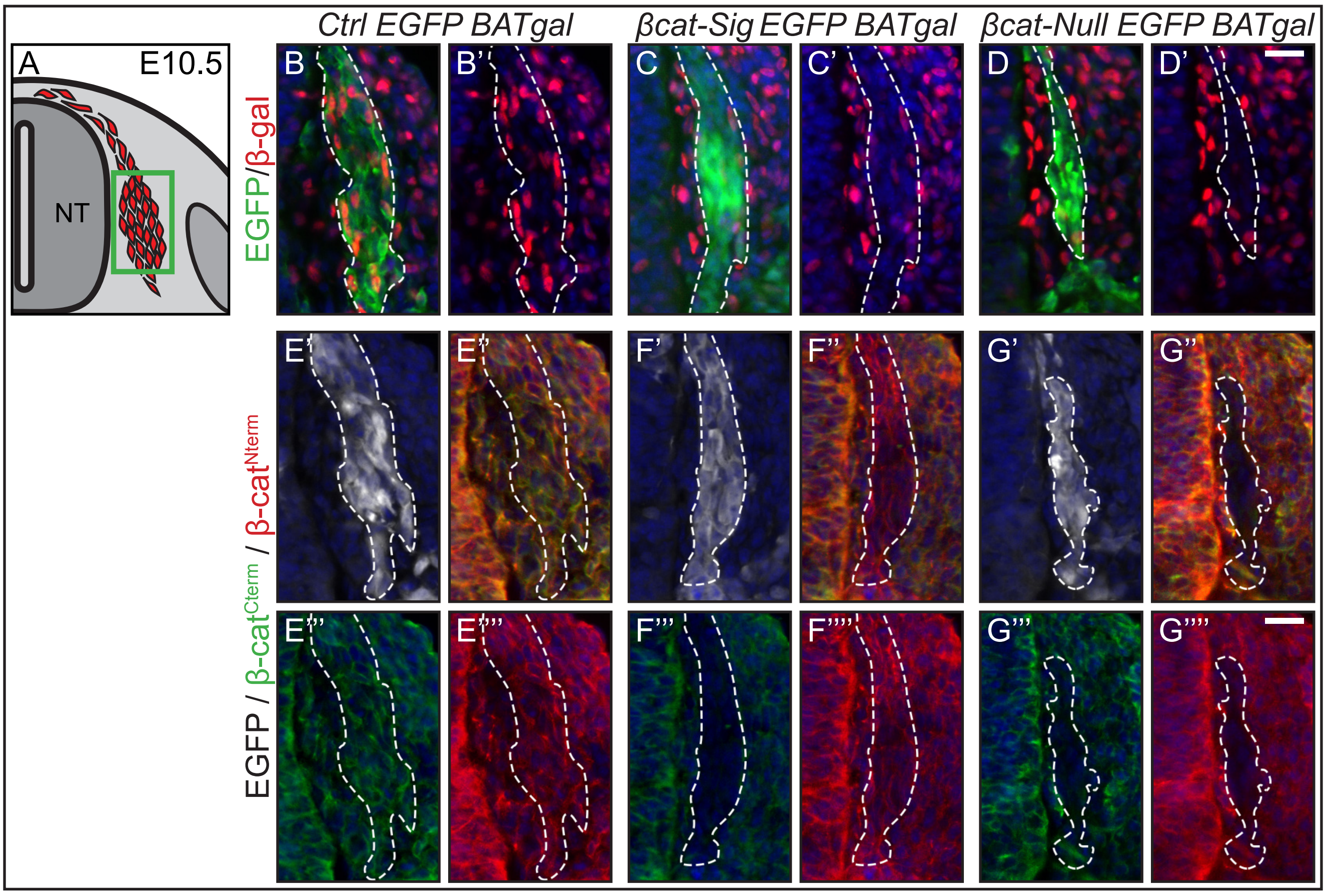

Additional file 1: Figure S2

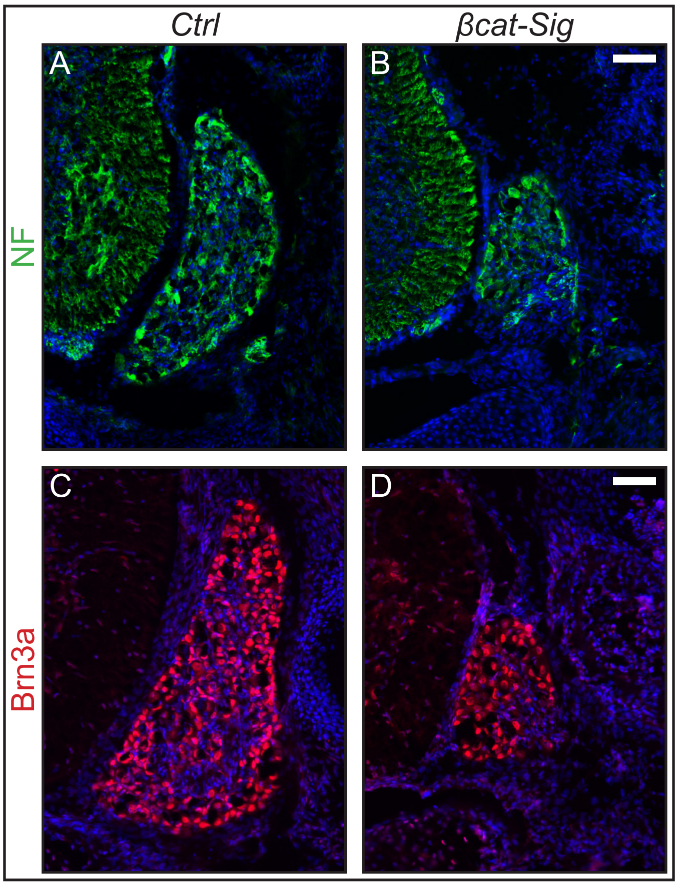

Additional file 1: Figure S3

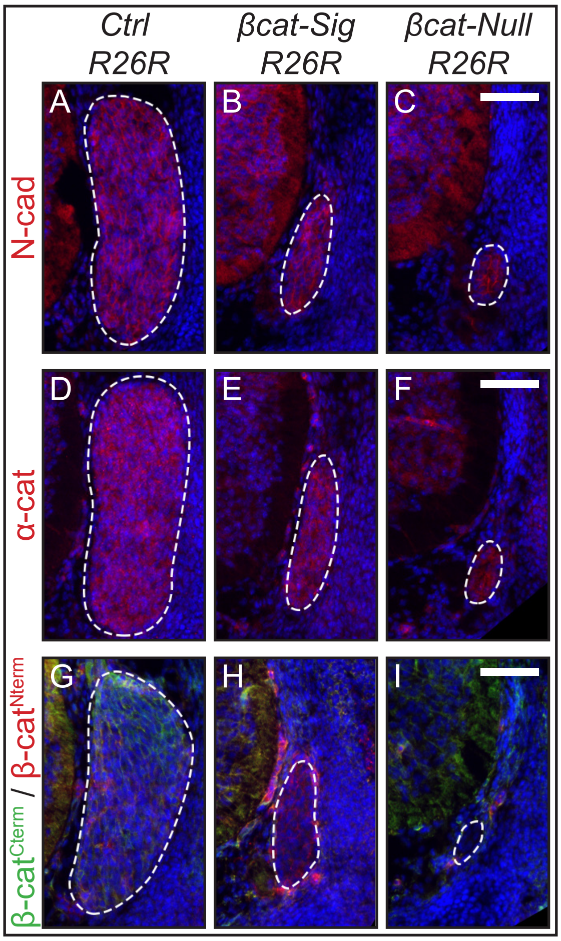

Additional file 1: Figure S4

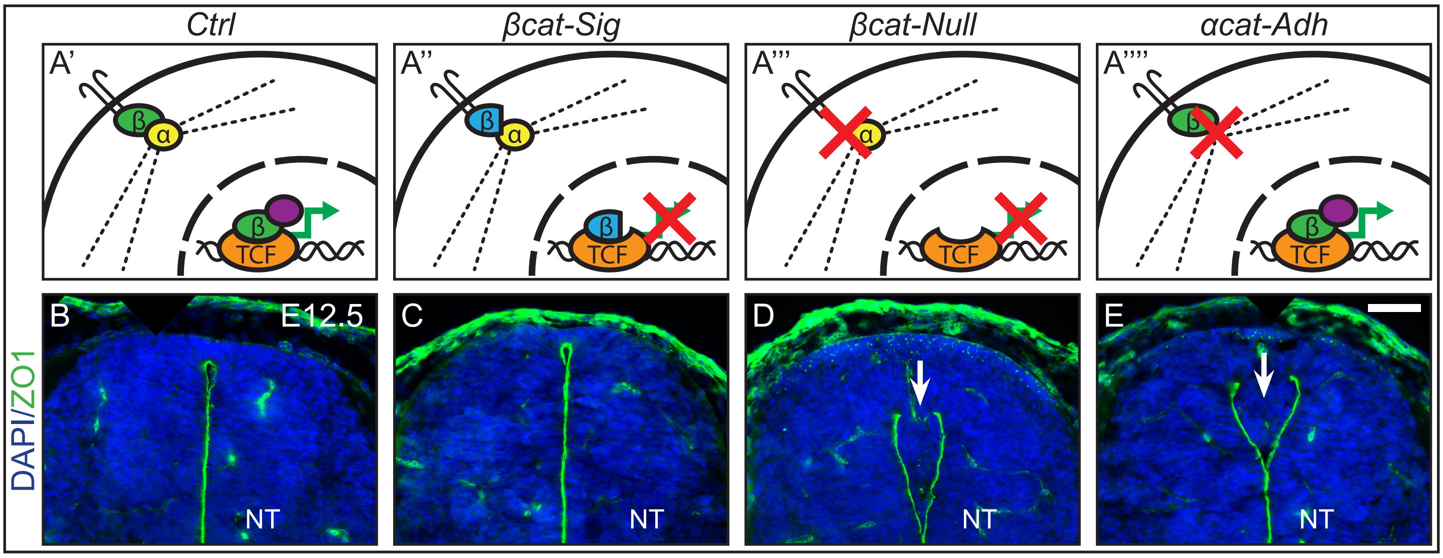

Additional file 1: Figure S5

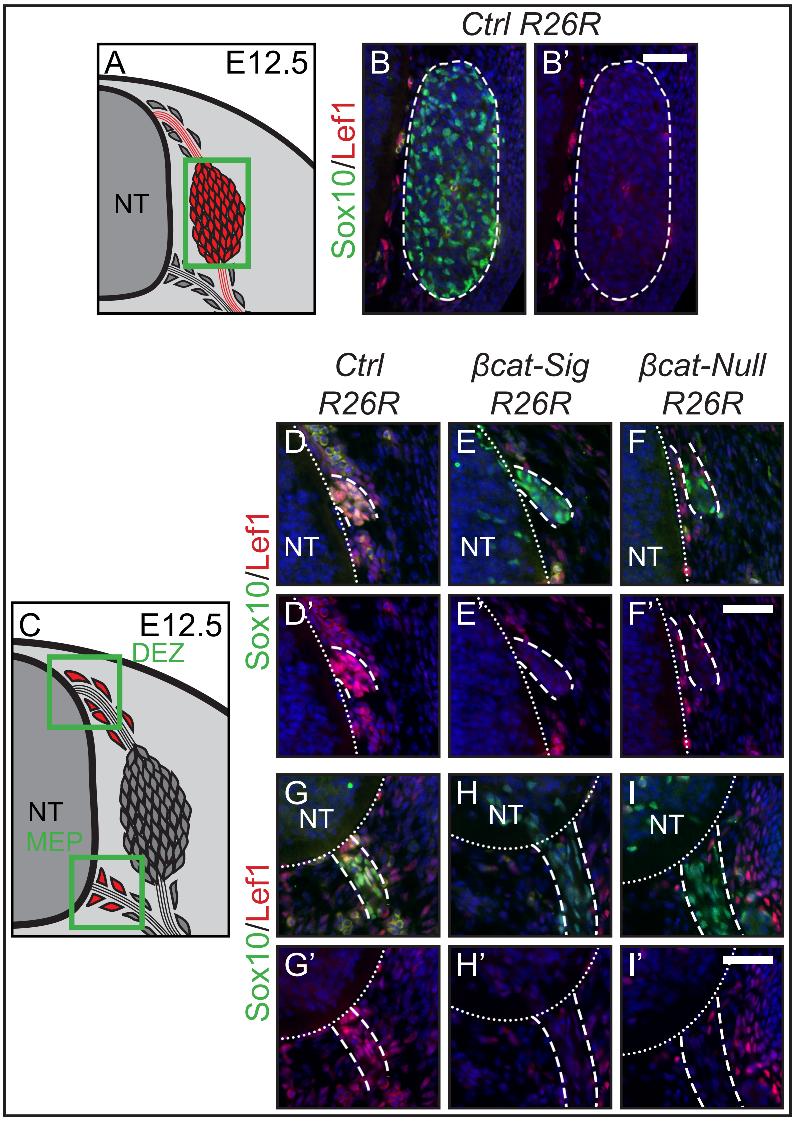

Additional file 1: Figure S6

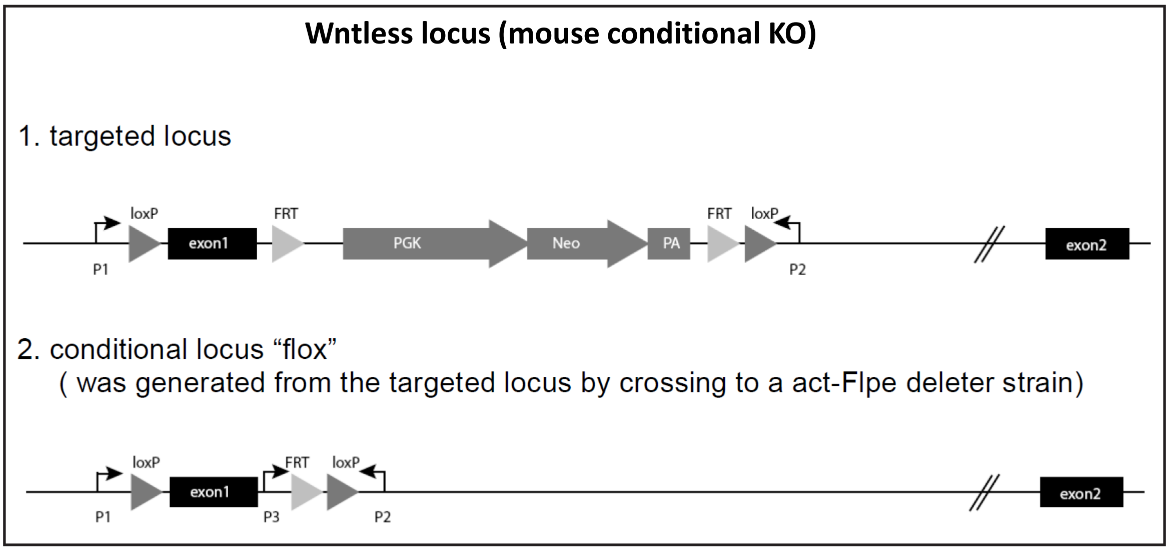

Additional file 1: Figure S7
